# A Statistical Evaluation of Methods of In-Vitro Growth Assessment for *Phyllosticta citricarpa*: Average Colony Diameter *vs*. Area

**DOI:** 10.1371/journal.pone.0170755

**Published:** 2017-01-26

**Authors:** Katherine E. Hendricks, Mary C. Christman, Pamela D. Roberts

**Affiliations:** 1 Department of Plant Pathology, University of Florida, Immokalee, Florida, United States of America; 2 Department of Statistics, University of Florida, Gainesville, Florida, United States of America; Universita degli Studi di Pisa, ITALY

## Abstract

Fungal growth inhibition on solid media has been historically measured and calculated based on the average of perpendicular diameter measurements of growth on fungicide amended media. We investigated the sensitivity of the calculated area (D_A_) and the measured area (M_A_) for assessing fungicide growth inhibition of the ascomycete, *Phyllosticta citricarpa* on solid media. Both the calculated, D_A_ and the actual measured area, M_A_ were adequate for distinguishing significant treatment effects of fungicide on fungal growth, however M_A_ was more sensitive at identifying significant differences between the controls and fungicide concentrations below 5 ppm.

## Introduction

Methods of filamentous fungal growth assessment depend upon the media in which the fungus is grown. Direct measurements of fungal growth are utilized when fungi are grown in liquid media and single time point measurements are required. Biomass production (fungal growth) can be measured directly from dry or wet weight assessment. In cases where there is difficulty in separating mycelia from media, indirect measures are utilized. Indirect measures of fungal growth include visual observation and measurement [1–6], indicator dye hydrolysis, increases in measurable component unique to the fungus such as growth linked enzymes—laccase, ergosterol and/or chitin [7–9] and absorbance [10–13]. Radial growth measurement is a popular indirect measure of filamentous growth on solid media [14–16] when assessing fungicidal properties of chemicals. Radial growth measurements are usually taken perpendicularly [17] and may be reported as the average of two or more pairs of measurements [6]. Measurements may be made utilizing pre-drawn lines prior to placement of the inoculating fungal plug or drawn at the time of assessment.

*Phyllosticta citricarpa* (McAlp.) is a phytopathogenic fungus causing significant economic losses in citrus. *P*. *citricarpa* is a filamentous fungus of the class ascomycetes, order Dothideales. Cultures grow well on agar media with optimal growth between 24 and 27°C [18–20]. Fungal growth on potato dextrose agar (PDA) is slow and characterized by compact dark pigmented mycelia with a lobate intermediate zone of white to grey colored mycelia and a distinct lobate translucent edge [19, 21]. In some *P*. *citricarpa* isolates, mycelia penetrate the media to form plaque-like growth [21].

**Fig 1:**
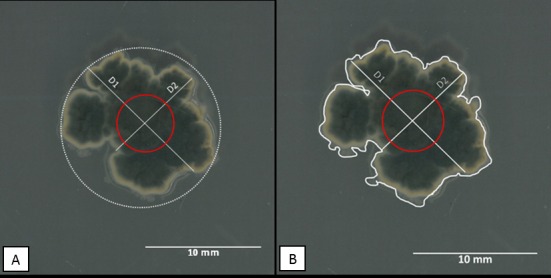
Day 7 growth of *Phyllosticta citricarpa* on unamended PDA. (A) Measurements D1 and D2 are the perpendicular length for estimating growth and used to compute calculated area (D_A_). The red circle is the approximate placement and size of the plug. (B) Measured area (M_A_) is represented by the area encompassed by the white outline of the colony.

The relationship between fungal colony diameter and actual growth is governed by the assumption that colony diameter is a good indicator of colony growth. For this assumption to hold true: (i) the rate of growth in all directions must be equal, such that measurements made perpendicular to each other will give the average of overall growth of the fungus and (ii) growth occurs only on the surface of the media or that growth into the media is minimal and does not affect horizontal growth. Because *P*. *citricarpa* does not conform to these parameters (Fig 1) we tested the hypotheses that calculated area (D_A_) using the averages of perpendicular diameter to predict inhibition will be less sensitive than the use of the measured area (M_A_).

## Material and Methods

### Fungal isolates

A selection of *P*. *citricarpa* isolates were obtained from citrus black spot lesions on fruits isolated between 2010 and 2013. Isolates were maintained on potato dextrose agar (PDA) at room temperature and under ambient light. These isolates were assumed to be clonal as there has been no evidence of both mating types in Florida to date [22] and lack of sexual structure produced in mating studies (data not shown). A total of 22 isolates were used (Table 1).

**Table 1:** List of *Phyllosticta citricarpa* isolates obtained from *Citrus sinensis* fruits in Florida and their characteristic included in this study.

Year isolated^a^	Location^b^	Isolate no.	Other name^c^	Symptoms^d^	ITS Sequence^e^	Yellow pigment^f^
2010	1	2010_01	FLGC1	Hard spot	yes	Present
2010	1	2010_03	FLGC3	Hard spot	yes	Present
2010	2	2010_04	FLGC4	Hard spot	no	Present
2010	2	2010_06	FLGC6	False melanose	no	Present
2010	2	2010_08	FLGC8	False melanose	no	Present
2010	2	2010_10	FLGC10	False melanose	no	Present
2011	3	2011_02	-	-	yes	Present
2011	2	2011_15	FLGC15	-	no	Present
2011	2	2011_63	-	False melanose	no	Present
2011	2	2011_67	-	Hard spot	no	Present
2011	2	2011_88	-	-	no	Present
2012	2	2012_051	-	Hard spot	no	Present
2012	2	2012_086	-	Hard spot	no	Present
2012	2	2012_117	-	Hard spot	no	Present
2012	2	2012_125	-	Hard spot	no	Present
2012	2	2012_135	-	Hard spot	no	Present
2013	3	2013_01	-	-	no	Present
2013	3	2013_03	-	-	no	Present
2013	3	2013_04	-	-	no	Present
2013	3	2013_11	-	-	no	Present
2013	3	2013_18	-	-	no	Present
2013	3	2013_20	-	-	no	Present

^a^Year fruit with symptoms were collected and isolates isolated from lesions.

^b^Unique locations (groves) from which diseased fruit were obtained.

^c^Other isolate identification codes, these were used in the study of copper on growth characteristics of *Phyllosticta citricarpa* [23].

^d^Lesion from which the fungus was isolated; a dash (-) indicates no information on lesion used for isolation, however isolations where typically made from hard-spots, false melanose and virulent spots.

^e^Sequencing of the ITS region for molecular identification using CITRI1/ITS4 primers [24].

^f^One of the characteristic features of *P*. *citricarpa* is the yellow pigment produces when cultured on oatmeal agar [21, 25].

### Fungal growth

Fungal growth was assessed using (i) that calculated area (D_A_) and (ii) measured colony area (M_A_) on Day 7, 14 and 21 on PDA with and without fungicide. Each isolate was grown on PDA with and without the demethylation inhibitor (DMI) fungicide, fenbuconazole (dissolved in acetone) to obtain a final concentration of 0.1, 1, 5, and 10 ppm (μg/ml). Controls were PDA and PDA amended with acetone (1 ml/L). Plates were maintained at 25°C for 21 days under a 12 hr light-dark cycle and scans of each plate were taken using a on days 7, 14, and 21 post inoculation. Scans were made using a Konica Minolta Bizhub C652 (Konica Minolta Business Solutions U.S.A., Inc.) and saved as JPEG images (600 x 600 dpi, 24 bit depth, file size ranged from 2.19 to 3.01 MB). Scans were analyzed using the National Institutes of Health ImageJ v1.4 software, measure tool (rsb.info.nih.gov/ij/index.html) [26]. Two replicated were carried out per isolate per fungicide concentration and the entire experiment was repeated twice.

Methods of measurement were compared to determine the differences in analyzed results and whether these differences changed the final conclusion made at the treatment level. In order to compare measured area (M_A_) to diameter (D), average diameter was converted to area of a circle,

$$D_{A}=\pi r^{2}=\pi {\left(\frac{AverageD}{2}\right)}^{2}$$ (1)

and then compared to measured area, M_A_. Inhibition ratios, Ratio_0_ and Ratio_A_ were calculated using the controls–PDA and PDA amended with acetone (1 ml/L) respectively using the formula:

$$Inhibition\;Ratio=\frac{\left(Average\;Control\;Area-Average\;Treatment\;Area\right)}{Average\;Control\;Area}$$ (2)

### Statistical analysis

Under normal circumstances an EC50 approach (the fungicide concertation required to reduce growth by 50% of the control) would be used to evaluate in vitro fungicide sensitivity of the fungus of interest. In this case, since the concentrations tested resulted either in no effect or greater than 50% reduction this approach was not used. Instead inhibition ratios were analyzed using a mixed model with fungicide concentration*day*method of fungal growth assessment (Media*Day*Method) as the fixed effect and repetition was the random effect. A Beta distribution for the ratio was assumed. Since the Beta distribution does not allow for values to be either 0 or 1, in the few instances where the observed ratio equaled 1 it was converted to 0.995 for analyses. All analysis was done in SAS 9.4 (SAS Institute Inc., NC) using PROC GLIMMIX. The Tukey method was used to control the family-wise error rate for multiple comparisons. A p-value ≤ 0.05 was considered statistically significant.

## Results and Discussion

**Fig 2:**
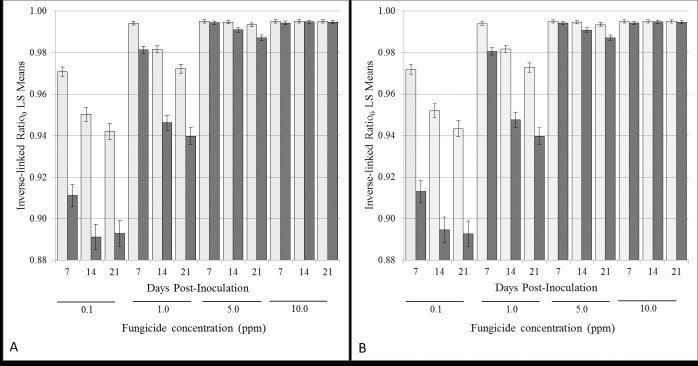
Comparison of LS-Means for the three-way interaction, Media*Day*Method, where dark bars represent measured Area (M_A_) and light bars represent area calculated from Diameter (D_A_). A. Inhibition ratios calculated using un-amended PDA as control (Ratio_0_) for each time point, Days 7, 14 and 21 for each fungicide level tested. B. Inhibition ratios calculated using acetone amended PDA as control (Ratio_A_) for each time point, Days 7, 14 and 21 for each fungicide level tested. The error-bars are the 95% confidence intervals for each mean.

**Fig 3:**
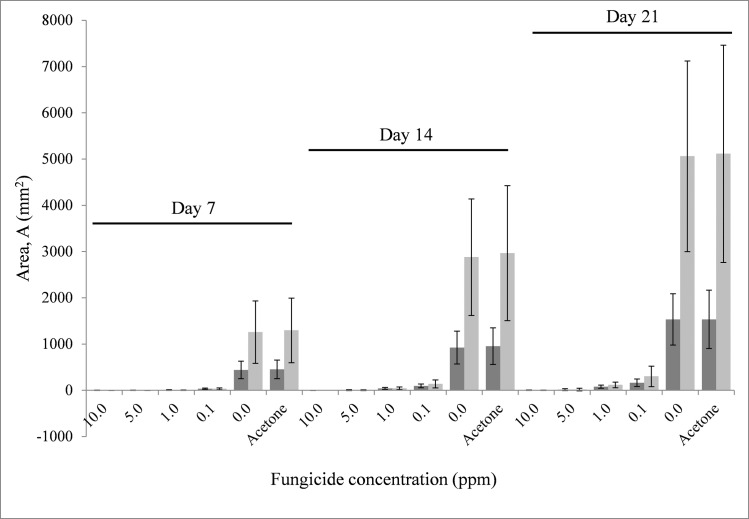
Area of fungal growth measured, M_A_ (dark bar) or calculated, D_A_ (light bar) from average diameter for Days 7, 14 and 21 for each fungicide level tested. The error-bars are the 95% confidence intervals for each mean.

In comparing methods of assessment of fungal growth on control media and media amended with fenbuconazole using inhibition ratios, the fixed effect Media*Day*Method was highly significant for Ratio_0_ and Ratio_A_, having p-values of p<0.0001 (for Ratio_0_, F_23, 2033_ = 259.27; for Ratio_A_, F_23, 2033_ = 273.35). Measurements of fungal growth inhibition by measured area (M_A_) were significantly different from calculated area, D_A_ at lower concentrations of fungicide (0.1 to 5 ppm) on all days measured with the exception of 5 ppm on Day 7 (Fig 2A). This can be attributed to the initial lag or inhibition in growth caused by higher concentration of the fungicide. There was no difference between methods used to assess fungicide growth inhibition for the 10 ppm on all days assessed (Fig 3; S1 Table) and this is attributed to complete inhibition of growth by the highest fungicide concentration. This was true for ratios calculated based on either PDA (Ratio_0_) or PDA amended with acetone (Ratio_A_) as controls (Fig 2A and 2B).

The measured area, M_A_ is a more sensitive means of distinguishing subtle changes in fungal growth inhibition at lower fungicide concentrations. This is further reflected in the ability of M_A_ to distinguish small changes in fungal growth inhibition between Day 7 and 14 (p = 0.0094), Day 7 and 21 (p<0.0001), and Day 14 and 21(p = 0.0262) at 5 ppm (Ratio_0_) which were indistinguishable using calculated area, D_A_ (p>0.05). Similar findings were found with ratios calculated using PDA amended with acetone (Ratio_A_) as control (Fig 2B–Measured Area, M_A_—Day 7 vs 14 (p = 0.0104), Day 7 vs 21 (p<0.0001) and Day 14 vs 21(p = 0.0314)).

The lowest fungicide concentration (0.1 ppm) inhibited growth by an average of 80% (85%—Day 7; 79%—Day 14 and 76% -Day 21; p<0.05) when using the diameter of fungal growth and an average of 91% (92%—Day 7; 90%—Day 14 and 89% -Day 21; p<0.05) when using area to measure fungal inhibition. Overall, in this study D_A_ and/or M_A_ was adequate to distinguish significant treatment effects of fungicide on fungal growth, however M_A_ was more sensitive.

In this study, analysis of area calculated from colony diameter (D_A_) and measured area (M_A_) were adequate to distinguish significant differences in fungicide growth inhibition for *Phyllosticta citricarpa*. However, measured area (M_A_) proved to be more sensitive in distinguishing subtle changes in growth at and below 5 ppm fenbuconazole between days measured. This was expected as calculating the area of a circle using average diameter overestimates the true area (Fig 1).

A drawback of using two-dimensional measures such as area or diameter is that the third-dimension of colony density is not taken into consideration [27]. Additionally, there is evidence to suggest that as fungi age there is a greater rate of hyphal extension which is not reflected in changes in colony diameter [27], however the impact of fenbuconazole on this phenomenon was not examined in this study.

## Supporting Information

S1 Table Data set for the in-vitro growth assessment for *Phyllosticta citricarpa*: Average Colony Diameter vs. Area. (PDF)
