# Supplementary material for: A Statistical Evaluation of Methods of In-Vitro Growth Assessment for Phyllosticta citricarpa: Average Colony Diameter vs. Area
Source: PLoS One. 2017 Jan 26;12(1):e0170755. doi: 10.1371/journal.pone.0170755 (PMC5268463; doi:10.1371/journal.pone.0170755)
Supplement: S1 Table — (PDF) [file pone.0170755.s001.pdf]

S1. Table. Data set for the in-vitro growth assessment for *Phyllosticta citricarpa:* Average Colony Diameter vs. Area

Day	Rep	Isolate no.	Fungicide ppm (µg/ml)	Plate	M_A	D_A
7	1	2010_01	0.0	1	547.56	1463.03
7	1	2010_01	0.0	2	552.05	1559.48
7	1	2010_01	0.1	1	36.20	37.83
7	1	2010_01	0.1	2	45.56	33.29
7	1	2010_01	1.0	1	13.13	2.81
7	1	2010_01	1.0	2	9.89	3.30
7	1	2010_01	5.0	1	0.49	0.05
7	1	2010_01	5.0	2	1.74	0.48
7	1	2010_01	10.0	1	0.00	0.00
7	1	2010_01	10.0	2	1.00	0.08
7	1	2010_01	A	1	580.09	1577.03
7	1	2010_01	A	2	540.02	1352.65
7	1	2010_03	0.0	1	319.88	806.76
7	1	2010_03	0.0	2	306.17	768.96
7	1	2010_03	0.1	1	50.03	61.93
7	1	2010_03	0.1	2	45.91	41.17
7	1	2010_03	1.0	1	0.74	0.06
7	1	2010_03	1.0	2	1.34	0.29
7	1	2010_03	5.0	1	0.71	0.00
7	1	2010_03	5.0	2	0.00	0.00
7	1	2010_03	10.0	1	0.00	0.00
7	1	2010_03	10.0	2	0.00	0.00
7	1	2010_03	A	1	394.12	955.53
7	1	2010_03	A	2	454.77	1202.57
7	1	2010_04	0.0	1	595.22	1799.77
7	1	2010_04	0.0	2	488.70	1378.19
7	1	2010_04	0.1	1	12.60	4.83
7	1	2010_04	0.1	2	18.99	5.68
7	1	2010_04	1.0	1	0.00	0.00
7	1	2010_04	1.0	2	0.00	0.00
7	1	2010_04	5.0	1	0.00	0.00
7	1	2010_04	5.0	2	0.00	0.00
7	1	2010_04	10.0	1	0.53	0.06
7	1	2010_04	10.0	2	0.28	0.01
7	1	2010_04	A	1	593.77	1708.47
7	1	2010_04	A	2	603.29	1827.70

7	1	2010_06	0.0	1	412.81	949.51
7	1	2010_06	0.0	2	424.16	1169.00
7	1	2010_06	0.1	1	28.23	21.65
7	1	2010_06	0.1	2	33.06	29.61
7	1	2010_06	1.0	1	13.67	3.94
7	1	2010_06	1.0	2	24.41	15.83
7	1	2010_06	5.0	1	0.00	0.00
7	1	2010_06	5.0	2	0.00	0.00
7	1	2010_06	10.0	1	0.00	0.00
7	1	2010_06	10.0	2	0.00	0.00
7	1	2010_06	A	1	238.15	672.88
7	1	2010_06	A	2	383.98	848.57
7	1	2010_08	0.0	1	456.07	1141.89
7	1	2010_08	0.0	2	581.37	1606.02
7	1	2010_08	0.1	1	34.18	25.61
7	1	2010_08	0.1	2	32.95	30.09
7	1	2010_08	1.0	1	9.53	2.49
7	1	2010_08	1.0	2	11.39	4.26
7	1	2010_08	5.0	1	0.00	0.00
7	1	2010_08	5.0	2	0.00	0.00
7	1	2010_08	10.0	1	0.00	0.00
7	1	2010_08	10.0	2	0.53	0.02
7	1	2010_08	A	1	507.56	1414.62
7	1	2010_08	A	2	489.17	1138.90
7	1	2010_10	0.0	1	243.56	483.83
7	1	2010_10	0.0	2	236.79	627.68
7	1	2010_10	0.1	1	20.85	16.69
7	1	2010_10	0.1	2	23.01	10.93
7	1	2010_10	1.0	1	5.63	0.31
7	1	2010_10	1.0	2	8.33	0.82
7	1	2010_10	5.0	1	0.00	0.00
7	1	2010_10	5.0	2	0.00	0.00
7	1	2010_10	10.0	1	0.00	0.00
7	1	2010_10	10.0	2	0.00	0.00
7	1	2010_10	A	1	132.47	340.12
7	1	2010_10	A	2	276.55	641.08
7	1	2011_02	0.0	1	551.54	2725.64
7	1	2011_02	0.0	2	491.51	1578.44
7	1	2011_02	0.1	1	34.55	27.62
7	1	2011_02	0.1	2	42.92	33.90
7	1	2011_02	1.0	1	10.26	2.99

7	1	2011_02	1.0	2	9.58	4.26
7	1	2011_02	5.0	1	0.00	0.00
7	1	2011_02	5.0	2	0.00	0.00
7	1	2011_02	10.0	1	1.01	0.03
7	1	2011_02	10.0	2	0.53	0.06
7	1	2011_02	A	1	516.35	1578.44
7	1	2011_02	A	2	638.64	2070.96
7	1	2011_15	0.0	1	274.45	690.00
7	1	2011_15	0.0	2	327.61	920.78
7	1	2011_15	0.1	1	29.77	17.80
7	1	2011_15	0.1	2	31.86	22.56
7	1	2011_15	1.0	1	10.31	3.98
7	1	2011_15	1.0	2	9.66	2.24
7	1	2011_15	5.0	1	0.86	0.05
7	1	2011_15	5.0	2	0.00	0.00
7	1	2011_15	10.0	1	0.00	0.00
7	1	2011_15	10.0	2	0.25	0.01
7	1	2011_15	A	1	568.41	1996.62
7	1	2011_15	A	2	450.67	1305.48
7	1	2011_63	0.0	1	531.81	1386.76
7	1	2011_63	0.0	2	478.75	1223.56
7	1	2011_63	0.1	1	61.03	75.58
7	1	2011_63	0.1	2	56.86	74.20
7	1	2011_63	1.0	1	27.32	20.35
7	1	2011_63	1.0	2	26.32	20.67
7	1	2011_63	5.0	1	0.00	0.00
7	1	2011_63	5.0	2	5.01	0.80
7	1	2011_63	10.0	1	0.00	0.00
7	1	2011_63	10.0	2	0.00	0.00
7	1	2011_63	A	1	408.09	1155.71
7	1	2011_63	A	2	481.57	1437.38
7	1	2011_67	0.0	1	651.44	1857.37
7	1	2011_67	0.0	2	587.63	1939.22
7	1	2011_67	0.1	1	34.01	27.06
7	1	2011_67	0.1	2	38.23	29.90
7	1	2011_67	1.0	1	17.59	8.40
7	1	2011_67	1.0	2	14.18	4.56
7	1	2011_67	5.0	1	0.01	0.06
7	1	2011_67	5.0	2	0.53	0.01
7	1	2011_67	10.0	1	0.00	0.00
7	1	2011_67	10.0	2	0.53	0.00

7	1	2011_67	A	1	554.86	1820.88
7	1	2011_67	A	2	591.62	1952.52
7	1	2011_88	0.0	1	1027.79	3499.39
7	1	2011_88	0.0	2	1061.35	3743.62
7	1	2011_88	0.1	1	26.59	18.63
7	1	2011_88	0.1	2	31.74	20.91
7	1	2011_88	1.0	1	18.75	12.19
7	1	2011_88	1.0	2	13.94	7.50
7	1	2011_88	5.0	1	0.00	0.00
7	1	2011_88	5.0	2	0.00	0.00
7	1	2011_88	10.0	1	0.00	0.00
7	1	2011_88	10.0	2	0.00	0.00
7	1	2011_88	A	1	1062.61	3263.40
7	1	2011_88	A	2	835.51	2769.30
7	1	2012_051	0.0	1	315.11	911.66
7	1	2012_051	0.0	2	270.04	792.73
7	1	2012_051	0.1	1	36.43	22.56
7	1	2012_051	0.1	2	39.90	29.90
7	1	2012_051	1.0	1	10.15	2.60
7	1	2012_051	1.0	2	11.51	2.72
7	1	2012_051	5.0	1	0.54	0.02
7	1	2012_051	5.0	2	0.00	0.00
7	1	2012_051	10.0	1	0.00	0.00
7	1	2012_051	10.0	2	1.51	0.08
7	1	2012_051	A	1	417.17	1238.48
7	1	2012_051	A	2	186.48	422.37
7	1	2012_086	0.0	1	538.13	1824.67
7	1	2012_086	0.0	2	562.06	1722.42
7	1	2012_086	0.1	1	35.04	33.29
7	1	2012_086	0.1	2	28.72	21.48
7	1	2012_086	1.0	1	4.17	0.92
7	1	2012_086	1.0	2	2.24	0.34
7	1	2012_086	5.0	1	0.28	0.03
7	1	2012_086	5.0	2	0.02	0.00
7	1	2012_086	10.0	1	0.00	0.00
7	1	2012_086	10.0	2	0.00	0.00
7	1	2012_086	A	1	514.82	1653.96
7	1	2012_086	A	2	414.50	1389.40
7	1	2012_117	0.0	1	617.30	1533.69
7	1	2012_117	0.0	2	675.96	1980.02
7	1	2012_117	0.1	1	44.21	32.88

7	1	2012_117	0.1	2	52.21	52.94
7	1	2012_117	1.0	1	26.28	15.00
7	1	2012_117	1.0	2	23.88	10.24
7	1	2012_117	5.0	1	8.08	4.56
7	1	2012_117	5.0	2	3.59	2.60
7	1	2012_117	10.0	1	0.02	0.00
7	1	2012_117	10.0	2	0.00	0.00
7	1	2012_117	A	1	469.14	1214.89
7	1	2012_117	A	2	499.60	1386.76
7	1	2012_125	0.0	1	429.13	1190.92
7	1	2012_125	0.0	2	400.71	1330.58
7	1	2012_125	0.1	1	39.37	39.26
7	1	2012_125	0.1	2	45.56	45.13
7	1	2012_125	1.0	1	16.71	6.33
7	1	2012_125	1.0	2	17.53	9.73
7	1	2012_125	5.0	1	0.00	0.00
7	1	2012_125	5.0	2	0.00	0.00
7	1	2012_125	10.0	1	0.00	0.00
7	1	2012_125	10.0	2	0.54	0.08
7	1	2012_125	A	1	372.23	1032.06
7	1	2012_125	A	2	448.65	1281.26
7	1	2012_135	0.0	1	185.60	502.33
7	1	2012_135	0.0	2	216.54	438.55
7	1	2012_135	0.1	1	38.34	32.37
7	1	2012_135	0.1	2	38.52	30.29
7	1	2012_135	1.0	1	18.65	11.46
7	1	2012_135	1.0	2	20.87	10.99
7	1	2012_135	5.0	1	0.53	0.07
7	1	2012_135	5.0	2	0.00	0.00
7	1	2012_135	10.0	1	0.00	0.00
7	1	2012_135	10.0	2	0.52	0.11
7	1	2012_135	A	1	198.12	408.64
7	1	2012_135	A	2	191.76	443.01
7	1	2013_01	0.0	1	299.43	709.69
7	1	2013_01	0.0	2	479.59	1778.03
7	1	2013_01	0.1	1	23.71	14.39
7	1	2013_01	0.1	2	32.08	31.37
7	1	2013_01	1.0	1	11.45	2.35
7	1	2013_01	1.0	2	12.54	3.91
7	1	2013_01	5.0	1	0.28	0.03
7	1	2013_01	5.0	2	0.00	0.00

7	1	2013_01	10.0	1	0.54	0.00
7	1	2013_01	10.0	2	0.00	0.00
7	1	2013_01	A	1	662.50	1980.81
7	1	2013_01	A	2	271.07	765.03
7	1	2013_03	0.0	1	532.43	1721.68
7	1	2013_03	0.0	2	445.59	1193.98
7	1	2013_03	0.1	1	18.69	13.01
7	1	2013_03	0.1	2	23.51	15.21
7	1	2013_03	1.0	1	14.36	7.89
7	1	2013_03	1.0	2	6.98	2.69
7	1	2013_03	5.0	1	1.75	0.16
7	1	2013_03	5.0	2	0.00	0.00
7	1	2013_03	10.0	1	0.53	0.06
7	1	2013_03	10.0	2	0.00	0.00
7	1	2013_03	A	1	597.19	1958.00
7	1	2013_03	A	2	536.38	1574.92
7	1	2013_04	0.0	1	534.33	1852.79
7	1	2013_04	0.0	2	608.93	1769.07
7	1	2013_04	0.1	1	39.96	37.18
7	1	2013_04	0.1	2	40.11	42.31
7	1	2013_04	1.0	1	12.03	5.23
7	1	2013_04	1.0	2	15.91	6.47
7	1	2013_04	5.0	1	7.53	1.70
7	1	2013_04	5.0	2	7.08	1.41
7	1	2013_04	10.0	1	0.00	0.00
7	1	2013_04	10.0	2	0.00	0.00
7	1	2013_04	A	1	283.13	830.60
7	1	2013_04	A	2	567.84	1845.93
7	1	2013_11	0.0	1	517.66	1417.29
7	1	2013_11	0.0	2	690.94	2174.66
7	1	2013_11	0.1	1	34.05	27.15
7	1	2013_11	0.1	2	45.47	42.08
7	1	2013_11	1.0	1	11.13	3.63
7	1	2013_11	1.0	2	6.85	1.89
7	1	2013_11	5.0	1	0.00	0.00
7	1	2013_11	5.0	2	0.00	0.00
7	1	2013_11	10.0	1	0.75	0.08
7	1	2013_11	10.0	2	0.00	0.00
7	1	2013_11	A	1	610.80	2219.52
7	1	2013_11	A	2	680.65	1955.65
7	1	2013_18	0.0	1	199.69	558.23

7	1	2013_18	0.0	2	510.68	1611.00
7	1	2013_18	0.1	1	28.57	28.09
7	1	2013_18	0.1	2	28.15	17.72
7	1	2013_18	1.0	1	15.41	4.71
7	1	2013_18	1.0	2	9.76	2.81
7	1	2013_18	5.0	1	0.00	0.00
7	1	2013_18	5.0	2	0.54	0.00
7	1	2013_18	10.0	1	0.00	0.00
7	1	2013_18	10.0	2	0.00	0.00
7	1	2013_18	A	1	473.21	1273.66
7	1	2013_18	A	2	504.37	1495.07
7	1	2013_20	0.0	1	495.48	1500.55
7	1	2013_20	0.0	2	574.58	1661.90
7	1	2013_20	0.1	1	41.66	36.42
7	1	2013_20	0.1	2	34.23	19.17
7	1	2013_20	1.0	1	14.76	3.60
7	1	2013_20	1.0	2	13.60	4.52
7	1	2013_20	5.0	1	0.02	0.22
7	1	2013_20	5.0	2	0.97	0.18
7	1	2013_20	10.0	1	0.99	0.06
7	1	2013_20	10.0	2	1.24	0.04
7	1	2013_20	A	1	483.08	1610.28
7	1	2013_20	A	2	583.87	1714.33
7	2	2010_01	0.0	1	232.57	564.95
7	2	2010_01	0.0	2	326.98	821.94
7	2	2010_01	0.1	1	28.10	25.43
7	2	2010_01	0.1	2	29.03	19.87
7	2	2010_01	1.0	1	5.28	5.47
7	2	2010_01	1.0	2	0.00	0.00
7	2	2010_01	5.0	1	0.00	0.00
7	2	2010_01	5.0	2	0.00	0.00
7	2	2010_01	10.0	1	0.00	0.00
7	2	2010_01	10.0	2	0.00	0.00
7	2	2010_01	A	1	433.79	1152.09
7	2	2010_01	A	2	334.00	1024.67
7	2	2010_03	0.0	1	665.67	1926.76
7	2	2010_03	0.0	2	423.98	1087.45
7	2	2010_03	0.1	1	65.38	65.61
7	2	2010_03	0.1	2	67.45	72.38
7	2	2010_03	1.0	1	0.00	0.00
7	2	2010_03	1.0	2	0.00	0.00

7	2	2010_03	5.0	1	0.00	0.00
7	2	2010_03	5.0	2	0.00	0.00
7	2	2010_03	10.0	1	0.00	0.00
7	2	2010_03	10.0	2	0.94	0.26
7	2	2010_03	A	1	393.51	925.09
7	2	2010_03	A	2	611.25	2570.60
7	2	2010_04	0.0	1	.	.
7	2	2010_04	0.0	2	102.97	174.83
7	2	2010_04	0.1	1	13.43	5.43
7	2	2010_04	0.1	2	21.12	13.79
7	2	2010_04	1.0	1	0.00	0.00
7	2	2010_04	1.0	2	0.00	0.00
7	2	2010_04	5.0	1	0.00	0.00
7	2	2010_04	5.0	2	0.00	0.00
7	2	2010_04	10.0	1	.	.
7	2	2010_04	10.0	2	0.00	0.00
7	2	2010_04	A	1	302.98	731.10
7	2	2010_04	A	2	.	.
7	2	2010_06	0.0	1	246.80	299.57
7	2	2010_06	0.0	2	360.13	513.91
7	2	2010_06	0.1	1	31.30	25.07
7	2	2010_06	0.1	2	39.27	39.26
7	2	2010_06	1.0	1	0.00	0.00
7	2	2010_06	1.0	2	0.00	0.00
7	2	2010_06	5.0	1	0.00	0.00
7	2	2010_06	5.0	2	0.00	0.00
7	2	2010_06	10.0	1	12.37	0.00
7	2	2010_06	10.0	2	0.00	0.00
7	2	2010_06	A	1	509.46	848.57
7	2	2010_06	A	2	401.67	586.21
7	2	2010_08	0.0	1	393.89	1124.58
7	2	2010_08	0.0	2	342.87	973.14
7	2	2010_08	0.1	1	29.24	21.57
7	2	2010_08	0.1	2	26.68	13.79
7	2	2010_08	1.0	1	0.00	0.00
7	2	2010_08	1.0	2	0.00	0.00
7	2	2010_08	5.0	1	0.00	0.00
7	2	2010_08	5.0	2	1.42	0.02
7	2	2010_08	10.0	1	0.25	0.00
7	2	2010_08	10.0	2	0.00	0.00
7	2	2010_08	A	1	397.13	1117.46

7	2	2010_08	A	2	426.99	1107.41
7	2	2010_10	0.0	1	223.83	503.12
7	2	2010_10	0.0	2	304.56	932.11
7	2	2010_10	0.1	1	32.67	30.19
7	2	2010_10	0.1	2	23.17	19.17
7	2	2010_10	1.0	1	2.06	0.11
7	2	2010_10	1.0	2	8.04	1.77
7	2	2010_10	5.0	1	0.00	0.00
7	2	2010_10	5.0	2	0.00	0.00
7	2	2010_10	10.0	1	0.00	0.00
7	2	2010_10	10.0	2	0.00	0.00
7	2	2010_10	A	1	302.05	727.27
7	2	2010_10	A	2	225.92	476.84
7	2	2011_02	0.0	1	551.74	1724.62
7	2	2011_02	0.0	2	688.50	2016.47
7	2	2011_02	0.1	1	52.22	50.27
7	2	2011_02	0.1	2	40.34	33.08
7	2	2011_02	1.0	1	0.25	0.05
7	2	2011_02	1.0	2	0.00	0.00
7	2	2011_02	5.0	1	1.17	0.07
7	2	2011_02	5.0	2	0.24	0.14
7	2	2011_02	10.0	1	1.91	0.31
7	2	2011_02	10.0	2	1.42	0.20
7	2	2011_02	A	1	790.55	2436.69
7	2	2011_02	A	2	747.06	2296.16
7	2	2011_15	0.0	1	374.06	996.50
7	2	2011_15	0.0	2	367.87	1219.22
7	2	2011_15	0.1	1	30.48	34.42
7	2	2011_15	0.1	2	29.66	28.18
7	2	2011_15	1.0	1	0.00	0.00
7	2	2011_15	1.0	2	0.00	0.00
7	2	2011_15	5.0	1	0.00	0.00
7	2	2011_15	5.0	2	0.00	0.00
7	2	2011_15	10.0	1	0.00	0.00
7	2	2011_15	10.0	2	0.21	0.13
7	2	2011_15	A	1	374.46	1034.34
7	2	2011_15	A	2	240.63	699.34
7	2	2011_63	0.0	1	240.31	489.70
7	2	2011_63	0.0	2	257.64	572.13
7	2	2011_63	0.1	1	29.78	26.60
7	2	2011_63	0.1	2	30.20	31.37

7	2	2011_63	1.0	1	0.00	0.00
7	2	2011_63	1.0	2	0.00	0.00
7	2	2011_63	5.0	1	0.00	0.00
7	2	2011_63	5.0	2	0.00	0.00
7	2	2011_63	10.0	1	0.00	0.00
7	2	2011_63	10.0	2	0.00	0.00
7	2	2011_63	A	1	314.35	871.97
7	2	2011_63	A	2	280.91	679.33
7	2	2011_67	0.0	1	307.28	843.42
7	2	2011_67	0.0	2	347.88	968.72
7	2	2011_67	0.1	1	38.00	24.37
7	2	2011_67	0.1	2	32.63	27.06
7	2	2011_67	1.0	1	0.00	0.00
7	2	2011_67	1.0	2	0.00	0.00
7	2	2011_67	5.0	1	0.00	0.00
7	2	2011_67	5.0	2	0.00	0.00
7	2	2011_67	10.0	1	0.00	0.00
7	2	2011_67	10.0	2	0.00	0.00
7	2	2011_67	A	1	188.45	387.42
7	2	2011_67	A	2	372.91	1107.41
7	2	2011_88	0.0	1	621.71	1849.74
7	2	2011_88	0.0	2	628.07	1805.04
7	2	2011_88	0.1	1	11.34	4.95
7	2	2011_88	0.1	2	14.80	8.81
7	2	2011_88	1.0	1	0.00	0.00
7	2	2011_88	1.0	2	0.00	0.00
7	2	2011_88	5.0	1	0.00	0.00
7	2	2011_88	5.0	2	0.00	0.00
7	2	2011_88	10.0	1	1.42	0.05
7	2	2011_88	10.0	2	0.00	0.00
7	2	2011_88	A	1	466.60	1187.86
7	2	2011_88	A	2	669.07	2179.62
7	2	2012_051	0.0	1	271.86	617.07
7	2	2012_051	0.0	2	276.47	919.17
7	2	2012_051	0.1	1	37.29	41.85
7	2	2012_051	0.1	2	27.74	21.73
7	2	2012_051	1.0	1	0.00	0.00
7	2	2012_051	1.0	2	0.66	0.10
7	2	2012_051	5.0	1	0.16	0.03
7	2	2012_051	5.0	2	1.36	0.02
7	2	2012_051	10.0	1	0.14	0.13

7	2	2012_051	10.0	2	0.15	0.01
7	2	2012_051	A	1	529.96	1364.41
7	2	2012_051	A	2	460.80	1111.55
7	2	2012_086	0.0	1	512.82	1547.61
7	2	2012_086	0.0	2	.	.
7	2	2012_086	0.1	1	21.40	12.57
7	2	2012_086	0.1	2	28.40	18.40
7	2	2012_086	1.0	1	11.30	2.43
7	2	2012_086	1.0	2	.	.
7	2	2012_086	5.0	1	0.15	0.06
7	2	2012_086	5.0	2	.	.
7	2	2012_086	10.0	1	2.13	0.13
7	2	2012_086	10.0	2	.	.
7	2	2012_086	A	1	545.42	1628.12
7	2	2012_086	A	2	.	.
7	2	2012_117	0.0	1	300.77	732.05
7	2	2012_117	0.0	2	280.21	785.76
7	2	2012_117	0.1	1	18.03	14.52
7	2	2012_117	0.1	2	18.08	9.79
7	2	2012_117	1.0	1	0.66	0.00
7	2	2012_117	1.0	2	0.00	0.00
7	2	2012_117	5.0	1	0.00	0.00
7	2	2012_117	5.0	2	0.66	0.04
7	2	2012_117	10.0	1	1.85	0.01
7	2	2012_117	10.0	2	1.84	0.10
7	2	2012_117	A	1	431.09	1227.28
7	2	2012_117	A	2	516.12	1526.76
7	2	2012_125	0.0	1	175.24	394.79
7	2	2012_125	0.0	2	187.80	394.79
7	2	2012_125	0.1	1	41.65	37.07
7	2	2012_125	0.1	2	39.21	30.09
7	2	2012_125	1.0	1	0.16	0.08
7	2	2012_125	1.0	2	2.38	0.02
7	2	2012_125	5.0	1	0.00	0.00
7	2	2012_125	5.0	2	2.60	0.30
7	2	2012_125	10.0	1	2.37	0.59
7	2	2012_125	10.0	2	0.12	0.11
7	2	2012_125	A	1	112.04	150.44
7	2	2012_125	A	2	227.43	543.67
7	2	2012_135	0.0	1	130.46	243.28
7	2	2012_135	0.0	2	.	.

7	2	2012_135	0.1	1	25.66	20.03
7	2	2012_135	0.1	2	21.36	14.79
7	2	2012_135	1.0	1	1.12	0.26
7	2	2012_135	1.0	2	.	.
7	2	2012_135	5.0	1	0.65	0.03
7	2	2012_135	5.0	2	.	.
7	2	2012_135	10.0	1	0.00	0.00
7	2	2012_135	10.0	2	.	.
7	2	2012_135	A	1	158.71	429.69
7	2	2012_135	A	2	.	.
7	2	2013_01	0.0	1	327.18	917.02
7	2	2013_01	0.0	2	350.20	811.30
7	2	2013_01	0.1	1	17.95	11.04
7	2	2013_01	0.1	2	56.95	50.39
7	2	2013_01	1.0	1	2.10	0.04
7	2	2013_01	1.0	2	0.15	0.06
7	2	2013_01	5.0	1	0.00	0.00
7	2	2013_01	5.0	2	2.84	0.05
7	2	2013_01	10.0	1	1.36	0.03
7	2	2013_01	10.0	2	1.12	0.06
7	2	2013_01	A	1	281.01	586.21
7	2	2013_01	A	2	185.99	338.82
7	2	2013_03	0.0	1	654.88	2248.85
7	2	2013_03	0.0	2	.	.
7	2	2013_03	0.1	1	17.45	12.32
7	2	2013_03	0.1	2	23.30	14.19
7	2	2013_03	1.0	1	11.27	7.16
7	2	2013_03	1.0	2	7.08	4.49
7	2	2013_03	5.0	1	3.59	0.16
7	2	2013_03	5.0	2	0.00	0.00
7	2	2013_03	10.0	1	3.85	0.08
7	2	2013_03	10.0	2	.	.
7	2	2013_03	A	1	462.21	1403.31
7	2	2013_03	A	2	.	.
7	2	2013_04	0.0	1	581.20	1573.51
7	2	2013_04	0.0	2	521.58	1503.30
7	2	2013_04	0.1	1	45.86	62.49
7	2	2013_04	0.1	2	28.90	21.81
7	2	2013_04	1.0	1	6.84	1.50
7	2	2013_04	1.0	2	2.84	0.59
7	2	2013_04	5.0	1	0.33	0.05

7	2	2013_04	5.0	2	0.00	0.00
7	2	2013_04	10.0	1	3.07	0.10
7	2	2013_04	10.0	2	1.11	0.38
7	2	2013_04	A	1	351.07	941.33
7	2	2013_04	A	2	445.56	1511.56
7	2	2013_11	0.0	1	363.38	1024.11
7	2	2013_11	0.0	2	306.81	776.84
7	2	2013_11	0.1	1	23.81	21.40
7	2	2013_11	0.1	2	38.46	33.59
7	2	2013_11	1.0	1	0.00	0.00
7	2	2013_11	1.0	2	1.12	0.00
7	2	2013_11	5.0	1	0.00	0.00
7	2	2013_11	5.0	2	0.00	0.00
7	2	2013_11	10.0	1	2.08	0.10
7	2	2013_11	10.0	2	0.15	0.00
7	2	2013_11	A	1	140.30	246.89
7	2	2013_11	A	2	487.30	1375.56
7	2	2013_18	0.0	1	535.12	1576.33
7	2	2013_18	0.0	2	358.50	871.44
7	2	2013_18	0.1	1	35.67	30.97
7	2	2013_18	0.1	2	36.01	30.97
7	2	2013_18	1.0	1	3.31	0.38
7	2	2013_18	1.0	2	0.00	0.00
7	2	2013_18	5.0	1	0.00	0.00
7	2	2013_18	5.0	2	2.10	0.26
7	2	2013_18	10.0	1	3.05	0.49
7	2	2013_18	10.0	2	4.61	0.01
7	2	2013_18	A	1	274.66	720.11
7	2	2013_18	A	2	261.57	720.11
7	2	2013_20	0.0	1	464.18	1386.76
7	2	2013_20	0.0	2	379.32	907.39
7	2	2013_20	0.1	1	47.42	48.27
7	2	2013_20	0.1	2	48.96	42.78
7	2	2013_20	1.0	1	0.00	0.00
7	2	2013_20	1.0	2	0.35	0.02
7	2	2013_20	5.0	1	3.84	0.05
7	2	2013_20	5.0	2	3.07	0.03
7	2	2013_20	10.0	1	0.00	0.00
7	2	2013_20	10.0	2	0.41	0.13
7	2	2013_20	A	1	524.53	1624.54
7	2	2013_20	A	2	437.18	1288.25

14	1	2010_01	0.0	1	787.26	2308.07
14	1	2010_01	0.0	2	898.57	2609.38
14	1	2010_01	0.1	1	100.97	158.14
14	1	2010_01	0.1	2	110.04	137.06
14	1	2010_01	1.0	1	52.95	49.02
14	1	2010_01	1.0	2	43.93	38.05
14	1	2010_01	5.0	1	32.05	43.36
14	1	2010_01	5.0	2	17.66	22.23
14	1	2010_01	10.0	1	0.61	0.00
14	1	2010_01	10.0	2	1.38	0.04
14	1	2010_01	A	1	1071.90	3171.91
14	1	2010_01	A	2	1027.75	3104.39
14	1	2010_03	0.0	1	818.93	2529.42
14	1	2010_03	0.0	2	828.61	2428.82
14	1	2010_03	0.1	1	120.98	235.33
14	1	2010_03	0.1	2	100.85	138.09
14	1	2010_03	1.0	1	35.54	24.37
14	1	2010_03	1.0	2	24.86	14.86
14	1	2010_03	5.0	1	0.00	0.00
14	1	2010_03	5.0	2	0.00	0.00
14	1	2010_03	10.0	1	0.00	0.00
14	1	2010_03	10.0	2	0.00	0.00
14	1	2010_03	A	1	1084.09	3231.08
14	1	2010_03	A	2	1429.83	4176.22
14	1	2010_04	0.0	1	1279.33	3993.84
14	1	2010_04	0.0	2	841.00	2428.82
14	1	2010_04	0.1	1	75.35	106.05
14	1	2010_04	0.1	2	100.72	131.10
14	1	2010_04	1.0	1	33.16	24.81
14	1	2010_04	1.0	2	28.24	20.51
14	1	2010_04	5.0	1	0.00	0.00
14	1	2010_04	5.0	2	0.00	0.00
14	1	2010_04	10.0	1	0.00	0.00
14	1	2010_04	10.0	2	0.00	0.00
14	1	2010_04	A	1	950.23	2551.76
14	1	2010_04	A	2	957.44	3055.21
14	1	2010_06	0.0	1	970.28	2643.90
14	1	2010_06	0.0	2	788.40	2312.33
14	1	2010_06	0.1	1	90.89	129.49
14	1	2010_06	0.1	2	97.44	141.66
14	1	2010_06	1.0	1	61.43	69.69

14	1	2010_06	1.0	2	79.14	120.96
14	1	2010_06	5.0	1	0.00	0.00
14	1	2010_06	5.0	2	0.86	0.03
14	1	2010_06	10.0	1	0.00	0.00
14	1	2010_06	10.0	2	0.00	0.00
14	1	2010_06	A	1	563.40	1639.58
14	1	2010_06	A	2	904.15	2570.60
14	1	2010_08	0.0	1	1002.02	3196.92
14	1	2010_08	0.0	2	1067.88	3030.77
14	1	2010_08	0.1	1	81.79	80.75
14	1	2010_08	0.1	2	65.69	79.96
14	1	2010_08	1.0	1	42.18	30.29
14	1	2010_08	1.0	2	49.45	37.18
14	1	2010_08	5.0	1	0.00	0.00
14	1	2010_08	5.0	2	0.00	0.00
14	1	2010_08	10.0	1	0.00	0.00
14	1	2010_08	10.0	2	0.00	0.00
14	1	2010_08	A	1	1013.58	3338.76
14	1	2010_08	A	2	762.28	2019.65
14	1	2010_10	0.0	1	562.46	1333.17
14	1	2010_10	0.0	2	592.56	1544.82
14	1	2010_10	0.1	1	72.22	84.79
14	1	2010_10	0.1	2	77.96	76.67
14	1	2010_10	1.0	1	32.30	32.78
14	1	2010_10	1.0	2	35.35	34.94
14	1	2010_10	5.0	1	2.32	0.05
14	1	2010_10	5.0	2	0.00	0.00
14	1	2010_10	10.0	1	0.00	0.00
14	1	2010_10	10.0	2	0.00	0.00
14	1	2010_10	A	1	345.41	702.62
14	1	2010_10	A	2	716.07	2338.83
14	1	2011_02	0.0	1	1144.57	4492.40
14	1	2011_02	0.0	2	1025.90	3235.11
14	1	2011_02	0.1	1	94.96	127.08
14	1	2011_02	0.1	2	98.53	140.19
14	1	2011_02	1.0	1	26.38	26.88
14	1	2011_02	1.0	2	27.04	15.55
14	1	2011_02	5.0	1	9.83	2.93
14	1	2011_02	5.0	2	0.00	0.00
14	1	2011_02	10.0	1	0.15	0.17
14	1	2011_02	10.0	2	0.12	0.02

14	1	2011_02	A	1	1188.70	3989.36
14	1	2011_02	A	2	1224.66	3923.58
14	1	2011_15	0.0	1	624.06	1594.68
14	1	2011_15	0.0	2	705.83	2533.88
14	1	2011_15	0.1	1	83.92	82.68
14	1	2011_15	0.1	2	91.62	127.88
14	1	2011_15	1.0	1	43.78	23.16
14	1	2011_15	1.0	2	53.00	48.40
14	1	2011_15	5.0	1	0.00	0.00
14	1	2011_15	5.0	2	0.00	0.00
14	1	2011_15	10.0	1	0.00	0.00
14	1	2011_15	10.0	2	0.00	0.00
14	1	2011_15	A	1	1114.47	3535.13
14	1	2011_15	A	2	888.74	2742.32
14	1	2011_63	0.0	1	1164.16	3606.09
14	1	2011_63	0.0	2	1144.33	3333.64
14	1	2011_63	0.1	1	165.39	288.63
14	1	2011_63	0.1	2	175.38	322.70
14	1	2011_63	1.0	1	85.12	127.88
14	1	2011_63	1.0	2	97.21	141.03
14	1	2011_63	5.0	1	12.64	6.74
14	1	2011_63	5.0	2	24.59	23.16
14	1	2011_63	10.0	1	0.00	0.00
14	1	2011_63	10.0	2	0.00	0.00
14	1	2011_63	A	1	1095.46	3341.83
14	1	2011_63	A	2	957.18	2972.50
14	1	2011_67	0.0	1	1523.72	4931.50
14	1	2011_67	0.0	2	1023.70	3138.06
14	1	2011_67	0.1	1	102.26	130.50
14	1	2011_67	0.1	2	106.38	122.91
14	1	2011_67	1.0	1	50.05	50.77
14	1	2011_67	1.0	2	57.32	50.14
14	1	2011_67	5.0	1	0.00	0.00
14	1	2011_67	5.0	2	0.00	0.00
14	1	2011_67	10.0	1	0.00	0.00
14	1	2011_67	10.0	2	0.00	0.00
14	1	2011_67	A	1	1346.40	4292.71
14	1	2011_67	A	2	970.59	3130.12
14	1	2011_88	0.0	1	1906.35	6546.84
14	1	2011_88	0.0	2	1967.67	6254.74
14	1	2011_88	0.1	1	86.55	114.04

14	1	2011_88	0.1	2	105.76	131.31
14	1	2011_88	1.0	1	81.00	108.43
14	1	2011_88	1.0	2	58.69	76.82
14	1	2011_88	5.0	1	0.27	0.00
14	1	2011_88	5.0	2	0.00	0.00
14	1	2011_88	10.0	1	0.00	0.00
14	1	2011_88	10.0	2	0.00	0.00
14	1	2011_88	A	1	1840.90	6535.37
14	1	2011_88	A	2	1755.35	5784.51
14	1	2012_051	0.0	1	732.80	2060.48
14	1	2012_051	0.0	2	635.85	1760.88
14	1	2012_051	0.1	1	81.05	112.34
14	1	2012_051	0.1	2	96.90	127.68
14	1	2012_051	1.0	1	49.01	56.35
14	1	2012_051	1.0	2	27.61	29.32
14	1	2012_051	5.0	1	23.63	11.88
14	1	2012_051	5.0	2	24.26	8.14
14	1	2012_051	10.0	1	0.00	0.00
14	1	2012_051	10.0	2	0.00	0.00
14	1	2012_051	A	1	885.45	2721.94
14	1	2012_051	A	2	500.09	1578.44
14	1	2012_086	0.0	1	909.87	3038.58
14	1	2012_086	0.0	2	915.29	2795.48
14	1	2012_086	0.1	1	106.35	164.90
14	1	2012_086	0.1	2	104.93	124.29
14	1	2012_086	1.0	1	39.98	34.00
14	1	2012_086	1.0	2	29.15	22.99
14	1	2012_086	5.0	1	0.00	0.00
14	1	2012_086	5.0	2	0.00	0.00
14	1	2012_086	10.0	1	0.00	0.00
14	1	2012_086	10.0	2	0.00	0.00
14	1	2012_086	A	1	883.69	2947.43
14	1	2012_086	A	2	842.90	2948.39
14	1	2012_117	0.0	1	1229.51	4042.15
14	1	2012_117	0.0	2	1490.98	4735.58
14	1	2012_117	0.1	1	104.86	162.86
14	1	2012_117	0.1	2	110.84	151.53
14	1	2012_117	1.0	1	47.03	57.15
14	1	2012_117	1.0	2	59.02	68.96
14	1	2012_117	5.0	1	35.53	27.06
14	1	2012_117	5.0	2	21.58	16.33

14	1	2012_117	10.0	1	0.00	0.00
14	1	2012_117	10.0	2	2.08	1.67
14	1	2012_117	A	1	1017.57	3130.12
14	1	2012_117	A	2	1019.20	3179.90
14	1	2012_125	0.0	1	874.84	2623.89
14	1	2012_125	0.0	2	864.69	2843.48
14	1	2012_125	0.1	1	98.71	127.68
14	1	2012_125	0.1	2	105.50	161.73
14	1	2012_125	1.0	1	42.87	36.32
14	1	2012_125	1.0	2	59.29	62.91
14	1	2012_125	5.0	1	0.00	0.00
14	1	2012_125	5.0	2	10.93	5.23
14	1	2012_125	10.0	1	0.00	0.00
14	1	2012_125	10.0	2	0.00	0.00
14	1	2012_125	A	1	798.73	2647.55
14	1	2012_125	A	2	948.44	3021.99
14	1	2012_135	0.0	1	453.52	1326.06
14	1	2012_135	0.0	2	462.42	1037.76
14	1	2012_135	0.1	1	83.01	95.38
14	1	2012_135	0.1	2	78.86	109.73
14	1	2012_135	1.0	1	60.14	67.20
14	1	2012_135	1.0	2	37.59	51.02
14	1	2012_135	5.0	1	11.53	5.03
14	1	2012_135	5.0	2	0.00	0.00
14	1	2012_135	10.0	1	0.00	0.00
14	1	2012_135	10.0	2	0.00	0.00
14	1	2012_135	A	1	457.75	980.90
14	1	2012_135	A	2	448.81	1074.63
14	1	2013_01	0.0	1	809.90	2408.77
14	1	2013_01	0.0	2	1056.18	3349.01
14	1	2013_01	0.1	1	74.72	94.86
14	1	2013_01	0.1	2	85.26	126.08
14	1	2013_01	1.0	1	43.99	39.93
14	1	2013_01	1.0	2	48.76	38.93
14	1	2013_01	5.0	1	0.00	0.00
14	1	2013_01	5.0	2	0.00	0.00
14	1	2013_01	10.0	1	0.00	0.00
14	1	2013_01	10.0	2	0.72	0.02
14	1	2013_01	A	1	1405.80	4474.59
14	1	2013_01	A	2	759.82	2460.37
14	1	2013_03	0.0	1	1148.35	3923.58

14	1	2013_03	0.0	2	1011.27	2948.39
14	1	2013_03	0.1	1	80.27	100.64
14	1	2013_03	0.1	2	84.91	105.32
14	1	2013_03	1.0	1	65.53	62.63
14	1	2013_03	1.0	2	48.70	36.21
14	1	2013_03	5.0	1	9.11	4.91
14	1	2013_03	5.0	2	7.26	1.67
14	1	2013_03	10.0	1	0.00	0.00
14	1	2013_03	10.0	2	0.45	0.01
14	1	2013_03	A	1	1093.26	3203.94
14	1	2013_03	A	2	912.27	2764.64
14	1	2013_04	0.0	1	1008.04	3409.80
14	1	2013_04	0.0	2	915.21	2765.57
14	1	2013_04	0.1	1	99.79	133.76
14	1	2013_04	0.1	2	152.91	136.23
14	1	2013_04	1.0	1	54.01	57.95
14	1	2013_04	1.0	2	96.19	63.76
14	1	2013_04	5.0	2	39.77	25.88
14	1	2013_04	5.0	2	68.94	24.28
14	1	2013_04	10.0	1	0.00	0.00
14	1	2013_04	10.0	2	16.81	3.70
14	1	2013_04	A	1	659.46	1820.88
14	1	2013_04	A	2	1192.42	3568.93
14	1	2013_11	0.0	1	978.58	3288.76
14	1	2013_11	0.0	2	1159.85	4189.97
14	1	2013_11	0.1	1	96.27	104.59
14	1	2013_11	0.1	2	102.18	117.09
14	1	2013_11	1.0	1	61.00	52.55
14	1	2013_11	1.0	2	59.21	62.63
14	1	2013_11	5.0	1	0.00	0.00
14	1	2013_11	5.0	2	0.00	0.00
14	1	2013_11	10.0	1	0.00	0.00
14	1	2013_11	10.0	2	0.00	0.00
14	1	2013_11	A	1	1085.26	3849.55
14	1	2013_11	A	2	1245.13	3401.52
14	1	2013_18	0.0	1	543.59	1696.04
14	1	2013_18	0.0	2	1101.98	3550.96
14	1	2013_18	0.1	1	91.23	115.56
14	1	2013_18	0.1	2	91.35	127.88
14	1	2013_18	1.0	1	53.40	50.27
14	1	2013_18	1.0	2	59.40	57.41

14	1	2013_18	5.0	1	1.92	0.10
14	1	2013_18	5.0	2	3.53	0.21
14	1	2013_18	10.0	1	0.00	0.00
14	1	2013_18	10.0	2	0.00	0.00
14	1	2013_18	A	1	853.09	2285.98
14	1	2013_18	A	2	1002.10	2981.21
14	1	2013_20	0.0	1	1065.79	3225.04
14	1	2013_20	0.0	2	1211.58	3780.58
14	1	2013_20	0.1	1	101.63	139.98
14	1	2013_20	0.1	2	91.16	114.80
14	1	2013_20	1.0	1	59.87	56.88
14	1	2013_20	1.0	2	63.62	63.90
14	1	2013_20	5.0	1	0.00	0.00
14	1	2013_20	5.0	2	0.00	0.00
14	1	2013_20	10.0	1	0.00	0.00
14	1	2013_20	10.0	2	0.00	0.00
14	1	2013_20	A	1	1166.24	4097.56
14	1	2013_20	A	2	1284.08	4267.20
14	2	2010_01	0.0	1	575.65	1511.56
14	2	2010_01	0.0	2	831.03	2617.54
14	2	2010_01	0.1	1	78.62	99.23
14	2	2010_01	0.1	2	81.27	127.68
14	2	2010_01	1.0	1	49.50	51.40
14	2	2010_01	1.0	2	27.73	32.67
14	2	2010_01	5.0	1	0.00	0.00
14	2	2010_01	5.0	2	0.00	0.00
14	2	2010_01	10.0	1	0.00	0.00
14	2	2010_01	10.0	2	0.00	0.00
14	2	2010_01	A	1	739.60	2050.84
14	2	2010_01	A	2	786.30	2740.46
14	2	2010_03	0.0	1	1837.62	6136.15
14	2	2010_03	0.0	2	1200.17	3856.15
14	2	2010_03	0.1	1	143.06	222.20
14	2	2010_03	0.1	2	166.95	267.64
14	2	2010_03	1.0	1	0.00	0.00
14	2	2010_03	1.0	2	0.97	1.06
14	2	2010_03	5.0	1	0.00	0.00
14	2	2010_03	5.0	2	0.00	0.00
14	2	2010_03	10.0	1	0.00	0.00
14	2	2010_03	10.0	2	0.00	0.00
14	2	2010_03	A	1	1006.17	2863.36

14	2	2010_03	A	2	1953.96	7777.20
14	2	2010_04	0.0	1	.	.
14	2	2010_04	0.0	2	305.36	843.93
14	2	2010_04	0.1	1	50.06	88.41
14	2	2010_04	0.1	2	60.91	81.55
14	2	2010_04	1.0	1	23.29	23.59
14	2	2010_04	1.0	2	15.00	15.76
14	2	2010_04	5.0	1	0.00	0.00
14	2	2010_04	5.0	2	0.00	0.00
14	2	2010_04	10.0	1	.	.
14	2	2010_04	10.0	2	0.00	0.00
14	2	2010_04	A	1	542.66	1420.63
14	2	2010_04	A	2	.	.
14	2	2010_06	0.0	1	567.70	1551.79
14	2	2010_06	0.0	2	488.14	1299.73
14	2	2010_06	0.1	1	91.81	131.10
14	2	2010_06	0.1	2	101.19	139.77
14	2	2010_06	1.0	1	53.53	52.94
14	2	2010_06	1.0	2	49.29	45.13
14	2	2010_06	5.0	1	0.40	0.00
14	2	2010_06	5.0	2	0.00	0.00
14	2	2010_06	10.0	1	1.04	0.13
14	2	2010_06	10.0	2	0.00	0.00
14	2	2010_06	A	1	746.76	1976.87
14	2	2010_06	A	2	655.63	1814.84
14	2	2010_08	0.0	1	759.03	2222.03
14	2	2010_08	0.0	2	577.04	1665.52
14	2	2010_08	0.1	1	87.23	121.74
14	2	2010_08	0.1	2	71.14	105.32
14	2	2010_08	1.0	1	17.25	5.77
14	2	2010_08	1.0	2	34.09	31.37
14	2	2010_08	5.0	1	0.00	0.00
14	2	2010_08	5.0	2	0.00	0.00
14	2	2010_08	10.0	1	0.00	0.00
14	2	2010_08	10.0	2	0.00	0.00
14	2	2010_08	A	1	809.49	2460.37
14	2	2010_08	A	2	893.25	2537.45
14	2	2010_10	0.0	1	501.67	1326.06
14	2	2010_10	0.0	2	863.27	2887.12
14	2	2010_10	0.1	1	80.66	109.73
14	2	2010_10	0.1	2	72.92	86.26

14	2	2010_10	1.0	1	27.21	25.52
14	2	2010_10	1.0	2	42.57	34.00
14	2	2010_10	5.0	1	1.42	0.69
14	2	2010_10	5.0	2	0.58	0.02
14	2	2010_10	10.0	1	0.00	0.00
14	2	2010_10	10.0	2	0.65	0.00
14	2	2010_10	A	1	738.38	2427.08
14	2	2010_10	A	2	476.90	1313.18
14	2	2011_02	0.0	1	989.56	3101.43
14	2	2011_02	0.0	2	1168.68	4128.25
14	2	2011_02	0.1	1	108.13	164.67
14	2	2011_02	0.1	2	95.52	156.37
14	2	2011_02	1.0	1	44.96	29.03
14	2	2011_02	1.0	2	37.55	36.10
14	2	2011_02	5.0	1	1.00	0.02
14	2	2011_02	5.0	2	2.48	0.00
14	2	2011_02	10.0	1	1.49	0.02
14	2	2011_02	10.0	2	0.53	0.03
14	2	2011_02	A	1	1399.23	4214.10
14	2	2011_02	A	2	1213.49	4171.64
14	2	2011_15	0.0	1	802.64	2207.02
14	2	2011_15	0.0	2	866.05	2965.74
14	2	2011_15	0.1	1	77.92	90.76
14	2	2011_15	0.1	2	77.41	91.61
14	2	2011_15	1.0	1	16.69	17.95
14	2	2011_15	1.0	2	27.89	19.87
14	2	2011_15	5.0	1	0.00	0.00
14	2	2011_15	5.0	2	0.00	0.00
14	2	2011_15	10.0	1	0.29	0.17
14	2	2011_15	10.0	2	0.00	0.00
14	2	2011_15	A	1	934.93	3027.84
14	2	2011_15	A	2	580.93	1636.71
14	2	2011_63	0.0	1	735.92	1910.45
14	2	2011_63	0.0	2	704.43	2165.58
14	2	2011_63	0.1	1	111.64	200.81
14	2	2011_63	0.1	2	115.06	214.60
14	2	2011_63	1.0	1	66.90	72.23
14	2	2011_63	1.0	2	74.85	122.13
14	2	2011_63	5.0	1	14.34	17.06
14	2	2011_63	5.0	2	0.00	0.00
14	2	2011_63	10.0	1	0.00	0.00

14	2	2011_63	10.0	2	0.00	0.00
14	2	2011_63	A	1	758.71	2217.02
14	2	2011_63	A	2	814.45	2534.78
14	2	2011_67	0.0	1	655.20	1905.03
14	2	2011_67	0.0	2	765.92	2424.46
14	2	2011_67	0.1	1	95.14	177.19
14	2	2011_67	0.1	2	93.31	148.92
14	2	2011_67	1.0	1	61.03	78.07
14	2	2011_67	1.0	2	55.49	60.68
14	2	2011_67	5.0	1	0.00	0.00
14	2	2011_67	5.0	2	0.00	0.00
14	2	2011_67	10.0	1	0.00	0.00
14	2	2011_67	10.0	2	0.00	0.00
14	2	2011_67	A	1	558.48	1618.12
14	2	2011_67	A	2	966.71	3064.04
14	2	2011_88	0.0	1	1492.56	4827.50
14	2	2011_88	0.0	2	1470.23	4905.39
14	2	2011_88	0.1	1	52.62	46.20
14	2	2011_88	0.1	2	47.74	49.27
14	2	2011_88	1.0	1	14.70	16.62
14	2	2011_88	1.0	2	26.38	21.81
14	2	2011_88	5.0	1	0.00	0.00
14	2	2011_88	5.0	2	0.00	0.00
14	2	2011_88	10.0	1	0.00	0.00
14	2	2011_88	10.0	2	0.00	0.00
14	2	2011_88	A	1	1145.24	3445.08
14	2	2011_88	A	2	1455.19	4419.04
14	2	2012_051	0.0	1	693.12	2252.21
14	2	2012_051	0.0	2	664.41	2424.46
14	2	2012_051	0.1	1	93.20	116.52
14	2	2012_051	0.1	2	80.83	107.88
14	2	2012_051	1.0	1	34.65	25.07
14	2	2012_051	1.0	2	35.24	26.60
14	2	2012_051	5.0	1	1.03	0.10
14	2	2012_051	5.0	2	0.54	0.03
14	2	2012_051	10.0	1	1.28	0.00
14	2	2012_051	10.0	2	0.00	0.00
14	2	2012_051	A	1	948.55	2625.71
14	2	2012_051	A	2	857.50	2315.74
14	2	2012_086	0.0	1	860.66	2434.94
14	2	2012_086	0.0	2	.	.

14	2	2012_086	0.1	1	61.91	82.03
14	2	2012_086	0.1	2	81.42	101.00
14	2	2012_086	1.0	1	25.33	16.62
14	2	2012_086	1.0	2	.	.
14	2	2012_086	5.0	1	0.00	0.00
14	2	2012_086	5.0	2	.	.
14	2	2012_086	10.0	1	1.26	0.09
14	2	2012_086	10.0	2	.	.
14	2	2012_086	A	1	864.90	2740.46
14	2	2012_086	A	2	.	.
14	2	2012_117	0.0	1	1232.41	4403.74
14	2	2012_117	0.0	2	673.87	2046.03
14	2	2012_117	0.1	1	64.12	77.29
14	2	2012_117	0.1	2	54.59	73.44
14	2	2012_117	1.0	1	17.16	10.24
14	2	2012_117	1.0	2	33.17	29.42
14	2	2012_117	5.0	1	0.00	0.00
14	2	2012_117	5.0	2	3.30	3.24
14	2	2012_117	10.0	1	0.00	0.00
14	2	2012_117	10.0	2	0.00	0.00
14	2	2012_117	A	1	1032.72	3483.68
14	2	2012_117	A	2	985.06	3200.93
14	2	2012_125	0.0	1	539.85	1530.22
14	2	2012_125	0.0	2	351.39	961.01
14	2	2012_125	0.1	1	83.43	103.33
14	2	2012_125	0.1	2	84.28	112.53
14	2	2012_125	1.0	1	52.07	55.68
14	2	2012_125	1.0	2	49.16	55.68
14	2	2012_125	5.0	1	1.92	0.02
14	2	2012_125	5.0	2	0.54	0.07
14	2	2012_125	10.0	1	2.97	0.17
14	2	2012_125	10.0	2	0.00	0.00
14	2	2012_125	A	1	355.11	821.94
14	2	2012_125	A	2	631.86	1969.78
14	2	2012_135	0.0	1	491.97	1254.13
14	2	2012_135	0.0	2	.	.
14	2	2012_135	0.1	1	65.89	91.61
14	2	2012_135	0.1	2	56.54	72.84
14	2	2012_135	1.0	1	19.30	13.79
14	2	2012_135	1.0	2	.	.
14	2	2012_135	5.0	1	16.15	9.73

14	2	2012_135	5.0	2	.	.
14	2	2012_135	10.0	1	3.99	4.79
14	2	2012_135	10.0	2	.	.
14	2	2012_135	A	1	499.23	1452.88
14	2	2012_135	A	2	.	.
14	2	2013_01	0.0	1	931.70	2568.80
14	2	2013_01	0.0	2	945.23	2764.64
14	2	2013_01	0.1	1	53.87	82.68
14	2	2013_01	0.1	2	114.69	171.57
14	2	2013_01	1.0	1	29.30	30.58
14	2	2013_01	1.0	2	27.52	17.95
14	2	2013_01	5.0	1	0.00	0.00
14	2	2013_01	5.0	2	0.00	0.00
14	2	2013_01	10.0	1	0.29	0.09
14	2	2013_01	10.0	2	0.00	0.00
14	2	2013_01	A	1	682.32	1936.88
14	2	2013_01	A	2	705.22	2056.46
14	2	2013_03	0.0	1	1172.79	4214.10
14	2	2013_03	0.0	2	.	.
14	2	2013_03	0.1	1	56.04	69.69
14	2	2013_03	0.1	2	69.79	85.28
14	2	2013_03	1.0	1	63.38	81.23
14	2	2013_03	1.0	2	30.78	28.18
14	2	2013_03	5.0	1	11.33	4.05
14	2	2013_03	5.0	2	9.00	5.68
14	2	2013_03	10.0	1	0.00	0.00
14	2	2013_03	10.0	2	.	.
14	2	2013_03	A	1	966.22	3802.41
14	2	2013_03	A	2	.	.
14	2	2013_04	0.0	1	1383.10	4665.10
14	2	2013_04	0.0	2	1187.01	4204.90
14	2	2013_04	0.1	1	107.02	194.83
14	2	2013_04	0.1	2	85.66	118.24
14	2	2013_04	1.0	1	53.32	75.28
14	2	2013_04	1.0	2	52.04	60.13
14	2	2013_04	5.0	1	0.00	0.00
14	2	2013_04	5.0	2	0.00	0.00
14	2	2013_04	10.0	1	0.00	0.00
14	2	2013_04	10.0	2	0.00	0.00
14	2	2013_04	A	1	910.55	2845.37
14	2	2013_04	A	2	926.05	2908.11

14	2	2013_11	0.0	1	685.90	2380.15
14	2	2013_11	0.0	2	746.34	2671.31
14	2	2013_11	0.1	1	79.77	100.47
14	2	2013_11	0.1	2	87.51	133.96
14	2	2013_11	1.0	1	49.86	50.27
14	2	2013_11	1.0	2	59.38	73.44
14	2	2013_11	5.0	1	0.04	0.02
14	2	2013_11	5.0	2	0.00	0.00
14	2	2013_11	10.0	1	0.00	0.00
14	2	2013_11	10.0	2	0.00	0.00
14	2	2013_11	A	1	482.05	1344.84
14	2	2013_11	A	2	1029.15	2870.00
14	2	2013_18	0.0	1	962.20	2930.14
14	2	2013_18	0.0	2	473.69	1394.03
14	2	2013_18	0.1	1	87.03	131.31
14	2	2013_18	0.1	2	90.67	104.05
14	2	2013_18	1.0	1	60.70	62.35
14	2	2013_18	1.0	2	52.59	51.78
14	2	2013_18	5.0	1	0.00	0.00
14	2	2013_18	5.0	2	1.00	0.15
14	2	2013_18	10.0	1	4.46	0.07
14	2	2013_18	10.0	2	0.00	0.00
14	2	2013_18	A	1	550.64	1530.22
14	2	2013_18	A	2	712.97	2494.78
14	2	2013_20	0.0	1	622.42	1712.13
14	2	2013_20	0.0	2	521.17	1526.76
14	2	2013_20	0.1	1	102.21	147.41
14	2	2013_20	0.1	2	97.89	145.48
14	2	2013_20	1.0	1	29.53	21.48
14	2	2013_20	1.0	2	46.73	35.68
14	2	2013_20	5.0	1	0.00	0.00
14	2	2013_20	5.0	2	0.46	0.01
14	2	2013_20	10.0	1	0.00	0.00
14	2	2013_20	10.0	2	1.42	0.26
14	2	2013_20	A	1	845.11	2696.11
14	2	2013_20	A	2	592.62	1562.28
21	1	2010_01	0.0	1	1451.53	5163.18
21	1	2010_01	0.0	2	1422.03	4357.99
21	1	2010_01	0.1	1	182.63	335.56
21	1	2010_01	0.1	2	178.88	295.90
21	1	2010_01	1.0	1	103.46	180.03

21	1	2010_01	1.0	2	106.78	148.71
21	1	2010_01	5.0	1	72.00	115.94
21	1	2010_01	5.0	2	85.37	146.77
21	1	2010_01	10.0	1	9.79	3.63
21	1	2010_01	10.0	2	3.15	0.66
21	1	2010_01	A	1	1791.16	6036.59
21	1	2010_01	A	2	1680.11	5243.73
21	1	2010_03	0.0	1	1300.10	4086.22
21	1	2010_03	0.0	2	1407.73	4462.75
21	1	2010_03	0.1	1	232.52	498.76
21	1	2010_03	0.1	2	208.23	370.86
21	1	2010_03	1.0	1	65.11	73.90
21	1	2010_03	1.0	2	57.10	67.06
21	1	2010_03	5.0	1	1.66	0.55
21	1	2010_03	5.0	2	10.91	0.00
21	1	2010_03	10.0	1	0.00	0.00
21	1	2010_03	10.0	2	0.00	0.00
21	1	2010_03	A	1	1752.28	5943.31
21	1	2010_03	A	2	2117.75	7127.07
21	1	2010_04	0.0	1	2115.67	6877.90
21	1	2010_04	0.0	2	978.69	2916.72
21	1	2010_04	0.1	1	123.22	211.50
21	1	2010_04	0.1	2	134.08	245.50
21	1	2010_04	1.0	1	96.08	120.76
21	1	2010_04	1.0	2	83.52	119.21
21	1	2010_04	5.0	1	12.19	15.98
21	1	2010_04	5.0	2	0.00	0.00
21	1	2010_04	10.0	1	0.00	0.00
21	1	2010_04	10.0	2	0.00	0.00
21	1	2010_04	A	1	1104.80	3786.03
21	1	2010_04	A	2	1263.68	4783.26
21	1	2010_06	0.0	1	1787.58	5380.66
21	1	2010_06	0.0	2	1420.66	4628.85
21	1	2010_06	0.1	1	143.91	237.51
21	1	2010_06	0.1	2	152.87	225.91
21	1	2010_06	1.0	1	107.45	197.81
21	1	2010_06	1.0	2	124.69	213.82
21	1	2010_06	5.0	1	0.00	0.00
21	1	2010_06	5.0	2	0.00	0.00
21	1	2010_06	10.0	1	0.00	0.00
21	1	2010_06	10.0	2	0.00	0.00

21	1	2010_06	A	1	890.44	2761.84
21	1	2010_06	A	2	1558.70	4797.99
21	1	2010_08	0.0	1	1609.28	6058.65
21	1	2010_08	0.0	2	1736.28	5491.74
21	1	2010_08	0.1	1	145.47	199.81
21	1	2010_08	0.1	2	126.30	217.21
21	1	2010_08	1.0	1	77.83	81.71
21	1	2010_08	1.0	2	87.93	115.94
21	1	2010_08	5.0	1	0.00	0.00
21	1	2010_08	5.0	2	0.00	0.00
21	1	2010_08	10.0	1	0.00	0.00
21	1	2010_08	10.0	2	0.00	0.00
21	1	2010_08	A	1	1562.37	5380.66
21	1	2010_08	A	2	912.55	2761.84
21	1	2010_10	0.0	1	1219.39	3717.64
21	1	2010_10	0.0	2	1050.79	3076.80
21	1	2010_10	0.1	1	0.00	0.00
21	1	2010_10	0.1	2	0.00	0.00
21	1	2010_10	1.0	1	0.00	0.00
21	1	2010_10	1.0	2	0.00	0.00
21	1	2010_10	5.0	1	0.00	0.00
21	1	2010_10	5.0	2	0.00	0.00
21	1	2010_10	10.0	1	0.00	0.00
21	1	2010_10	10.0	2	0.00	0.00
21	1	2010_10	A	1	717.16	1802.03
21	1	2010_10	A	2	1210.77	4614.39
21	1	2011_02	0.0	1	1999.77	7052.45
21	1	2011_02	0.0	2	1759.15	5850.76
21	1	2011_02	0.1	1	146.94	238.61
21	1	2011_02	0.1	2	170.85	348.34
21	1	2011_02	1.0	1	69.10	106.41
21	1	2011_02	1.0	2	45.70	48.89
21	1	2011_02	5.0	1	43.36	36.64
21	1	2011_02	5.0	2	6.59	3.46
21	1	2011_02	10.0	1	0.59	0.00
21	1	2011_02	10.0	2	1.32	0.07
21	1	2011_02	A	1	1863.40	6479.60
21	1	2011_02	A	2	1963.08	7025.68
21	1	2011_15	0.0	1	1124.54	3330.57
21	1	2011_15	0.0	2	1159.10	4285.74
21	1	2011_15	0.1	1	119.09	192.36

21	1	2011_15	0.1	2	141.66	238.61
21	1	2011_15	1.0	1	79.15	89.42
21	1	2011_15	1.0	2	81.55	106.41
21	1	2011_15	5.0	1	11.36	6.42
21	1	2011_15	5.0	2	54.80	54.76
21	1	2011_15	10.0	1	0.00	0.00
21	1	2011_15	10.0	2	0.00	0.00
21	1	2011_15	A	1	2061.86	6876.43
21	1	2011_15	A	2	1328.59	4299.68
21	1	2011_63	0.0	1	1825.38	6317.98
21	1	2011_63	0.0	2	1813.95	6789.99
21	1	2011_63	0.1	1	226.07	475.29
21	1	2011_63	0.1	2	240.33	472.21
21	1	2011_63	1.0	1	120.59	207.39
21	1	2011_63	1.0	2	122.32	226.98
21	1	2011_63	5.0	1	58.40	68.96
21	1	2011_63	5.0	2	61.16	92.97
21	1	2011_63	10.0	1	0.00	0.00
21	1	2011_63	10.0	2	0.00	0.00
21	1	2011_63	A	1	1796.34	6443.98
21	1	2011_63	A	2	1751.89	5966.56
21	1	2011_67	0.0	1	2299.41	8390.64
21	1	2011_67	0.0	2	1702.63	5783.17
21	1	2011_67	0.1	1	169.14	299.57
21	1	2011_67	0.1	2	148.41	213.82
21	1	2011_67	1.0	1	103.00	135.19
21	1	2011_67	1.0	2	84.30	98.17
21	1	2011_67	5.0	1	0.00	0.00
21	1	2011_67	5.0	2	0.00	0.00
21	1	2011_67	10.0	1	0.00	0.00
21	1	2011_67	10.0	2	0.00	0.00
21	1	2011_67	A	1	1984.51	7904.33
21	1	2011_67	A	2	1283.81	4299.68
21	1	2011_88	0.0	1	2732.32	10057.17
21	1	2011_88	0.0	2	2769.00	8703.60
21	1	2011_88	0.1	1	138.24	214.86
21	1	2011_88	0.1	2	141.09	264.46
21	1	2011_88	1.0	1	117.20	202.83
21	1	2011_88	1.0	2	99.58	179.32
21	1	2011_88	5.0	1	0.58	0.00
21	1	2011_88	5.0	2	5.03	2.84

21	1	2011_88	10.0	1	0.00	0.00
21	1	2011_88	10.0	2	0.00	0.00
21	1	2011_88	A	1	2650.91	8363.06
21	1	2011_88	A	2	2537.66	9386.19
21	1	2012_051	0.0	1	1056.62	3151.98
21	1	2012_051	0.0	2	950.98	2965.74
21	1	2012_051	0.1	1	136.51	230.73
21	1	2012_051	0.1	2	144.73	252.78
21	1	2012_051	1.0	1	92.30	147.63
21	1	2012_051	1.0	2	93.05	151.31
21	1	2012_051	5.0	1	50.54	42.78
21	1	2012_051	5.0	2	.	.
21	1	2012_051	10.0	1	0.00	0.00
21	1	2012_051	10.0	2	0.00	0.00
21	1	2012_051	A	1	1449.23	4992.68
21	1	2012_051	A	2	901.59	3189.90
21	1	2012_086	0.0	1	1664.85	5490.43
21	1	2012_086	0.0	2	1444.84	4720.95
21	1	2012_086	0.1	1	181.39	340.12
21	1	2012_086	0.1	2	183.29	326.21
21	1	2012_086	1.0	1	103.50	202.83
21	1	2012_086	1.0	2	74.68	97.47
21	1	2012_086	5.0	1	0.00	0.00
21	1	2012_086	5.0	2	10.35	5.35
21	1	2012_086	10.0	1	1.09	0.06
21	1	2012_086	10.0	2	0.00	0.00
21	1	2012_086	A	1	1426.60	4328.78
21	1	2012_086	A	2	1384.60	4388.46
21	1	2012_117	0.0	1	1945.68	5943.31
21	1	2012_117	0.0	2	2520.32	7825.73
21	1	2012_117	0.1	1	175.36	334.59
21	1	2012_117	0.1	2	158.71	265.62
21	1	2012_117	1.0	1	92.04	168.33
21	1	2012_117	1.0	2	120.65	171.10
21	1	2012_117	5.0	1	71.12	103.51
21	1	2012_117	5.0	2	61.16	73.29
21	1	2012_117	10.0	1	10.60	25.43
21	1	2012_117	10.0	2	24.95	20.43
21	1	2012_117	A	1	1643.73	5270.72
21	1	2012_117	A	2	1546.31	5131.39
21	1	2012_125	0.0	1	1481.46	4929.01

21	1	2012_125	0.0	2	1446.76	5019.01
21	1	2012_125	0.1	1	149.98	274.35
21	1	2012_125	0.1	2	182.98	315.42
21	1	2012_125	1.0	1	70.45	92.97
21	1	2012_125	1.0	2	108.47	181.22
21	1	2012_125	5.0	1	3.47	0.18
21	1	2012_125	5.0	2	24.10	21.16
21	1	2012_125	10.0	1	0.00	0.00
21	1	2012_125	10.0	2	0.00	0.00
21	1	2012_125	A	1	1315.95	4357.99
21	1	2012_125	A	2	1497.02	5557.61
21	1	2012_135	0.0	1	740.18	2426.20
21	1	2012_135	0.0	2	732.59	1997.41
21	1	2012_135	0.1	1	.	.
21	1	2012_135	0.1	2	123.69	199.81
21	1	2012_135	1.0	1	91.60	138.72
21	1	2012_135	1.0	2	65.66	98.17
21	1	2012_135	5.0	1	25.72	26.60
21	1	2012_135	5.0	2	0.00	0.00
21	1	2012_135	10.0	1	0.00	0.00
21	1	2012_135	10.0	2	0.00	0.00
21	1	2012_135	A	1	803.04	2632.98
21	1	2012_135	A	2	719.31	2007.72
21	1	2013_01	0.0	1	1702.10	5566.87
21	1	2013_01	0.0	2	1824.35	6576.99
21	1	2013_01	0.1	1	132.45	171.10
21	1	2013_01	0.1	2	153.46	269.09
21	1	2013_01	1.0	1	86.52	101.18
21	1	2013_01	1.0	2	85.03	120.96
21	1	2013_01	5.0	1	0.00	0.00
21	1	2013_01	5.0	2	0.00	0.00
21	1	2013_01	10.0	1	0.00	0.00
21	1	2013_01	10.0	2	0.00	0.00
21	1	2013_01	A	1	1879.19	6272.97
21	1	2013_01	A	2	1454.85	4862.04
21	1	2013_03	0.0	1	1876.00	5549.69
21	1	2013_03	0.0	2	1883.96	6009.08
21	1	2013_03	0.1	1	142.27	238.06
21	1	2013_03	0.1	2	158.23	288.93
21	1	2013_03	1.0	1	150.99	233.43
21	1	2013_03	1.0	2	82.37	117.67

21	1	2013_03	5.0	1	31.83	26.79
21	1	2013_03	5.0	2	28.48	23.07
21	1	2013_03	10.0	1	0.54	0.07
21	1	2013_03	10.0	2	1.04	0.16
21	1	2013_03	A	1	1878.60	5764.31
21	1	2013_03	A	2	1426.19	4505.47
21	1	2013_04	0.0	1	1728.09	6218.34
21	1	2013_04	0.0	2	1170.53	3749.04
21	1	2013_04	0.1	1	170.15	293.77
21	1	2013_04	0.1	2	162.40	271.42
21	1	2013_04	1.0	1	110.22	157.70
21	1	2013_04	1.0	2	141.81	205.87
21	1	2013_04	5.0	1	70.18	64.75
21	1	2013_04	5.0	2	72.81	88.08
21	1	2013_04	10.0	1	0.00	0.00
21	1	2013_04	10.0	2	38.10	27.99
21	1	2013_04	A	1	801.94	2298.71
21	1	2013_04	A	2	1846.47	6559.75
21	1	2013_11	0.0	1	1701.59	6141.71
21	1	2013_11	0.0	2	1938.69	7166.03
21	1	2013_11	0.1	1	148.95	241.08
21	1	2013_11	0.1	2	166.09	259.87
21	1	2013_11	1.0	1	112.68	145.05
21	1	2013_11	1.0	2	114.51	155.92
21	1	2013_11	5.0	1	5.67	2.11
21	1	2013_11	5.0	2	2.52	0.87
21	1	2013_11	10.0	1	0.00	0.00
21	1	2013_11	10.0	2	0.00	0.00
21	1	2013_11	A	1	1897.00	6920.60
21	1	2013_11	A	2	2063.37	6533.93
21	1	2013_18	0.0	1	1094.97	3541.46
21	1	2013_18	0.0	2	1730.25	5650.49
21	1	2013_18	0.1	1	155.46	254.47
21	1	2013_18	0.1	2	170.44	267.64
21	1	2013_18	1.0	1	103.90	136.85
21	1	2013_18	1.0	2	105.46	164.22
21	1	2013_18	5.0	1	20.42	11.46
21	1	2013_18	5.0	2	15.48	7.60
21	1	2013_18	10.0	1	0.00	0.00
21	1	2013_18	10.0	2	1.23	0.79
21	1	2013_18	A	1	1006.35	2787.99

21	1	2013_18	A	2	1356.05	4465.11
21	1	2013_20	0.0	1	1763.21	5658.49
21	1	2013_20	0.0	2	2013.34	6299.67
21	1	2013_20	0.1	1	165.64	288.32
21	1	2013_20	0.1	2	144.11	204.09
21	1	2013_20	1.0	1	108.38	153.28
21	1	2013_20	1.0	2	109.19	163.54
21	1	2013_20	5.0	1	46.17	38.93
21	1	2013_20	5.0	2	39.12	50.01
21	1	2013_20	10.0	1	0.00	0.00
21	1	2013_20	10.0	2	1.07	0.02
21	1	2013_20	A	1	1974.31	6876.43
21	1	2013_20	A	2	2163.80	7536.81
21	2	2010_01	0.0	1	1023.80	2793.61
21	2	2010_01	0.0	2	1431.24	4587.94
21	2	2010_01	0.1	1	146.79	249.13
21	2	2010_01	0.1	2	149.59	275.82
21	2	2010_01	1.0	1	98.10	154.38
21	2	2010_01	1.0	2	68.11	84.30
21	2	2010_01	5.0	1	0.00	0.00
21	2	2010_01	5.0	2	0.00	0.00
21	2	2010_01	10.0	1	0.00	0.00
21	2	2010_01	10.0	2	0.00	0.00
21	2	2010_01	A	1	1046.79	3376.76
21	2	2010_01	A	2	1294.96	4838.59
21	2	2010_03	0.0	1	3043.74	10511.91
21	2	2010_03	0.0	2	1709.93	5886.06
21	2	2010_03	0.1	1	285.91	653.25
21	2	2010_03	0.1	2	314.31	735.42
21	2	2010_03	1.0	1	10.84	7.45
21	2	2010_03	1.0	2	6.58	5.23
21	2	2010_03	5.0	1	0.00	0.00
21	2	2010_03	5.0	2	0.00	0.00
21	2	2010_03	10.0	1	0.00	0.00
21	2	2010_03	10.0	2	0.00	0.00
21	2	2010_03	A	1	1291.19	4139.65
21	2	2010_03	A	2	2988.83	11028.76
21	2	2010_04	0.0	1	.	.
21	2	2010_04	0.0	2	637.15	1907.35
21	2	2010_04	0.1	1	94.21	162.86
21	2	2010_04	0.1	2	117.49	186.51

21	2	2010_04	1.0	1	51.11	85.77
21	2	2010_04	1.0	2	55.59	67.49
21	2	2010_04	5.0	1	0.00	0.00
21	2	2010_04	5.0	2	0.00	0.00
21	2	2010_04	10.0	1	.	.
21	2	2010_04	10.0	2	0.00	0.00
21	2	2010_04	A	1	606.16	1849.74
21	2	2010_04	A	2	.	.
21	2	2010_06	0.0	1	1007.11	2817.08
21	2	2010_06	0.0	2	726.54	1996.62
21	2	2010_06	0.1	1	143.96	245.78
21	2	2010_06	0.1	2	165.78	297.12
21	2	2010_06	1.0	1	84.09	127.08
21	2	2010_06	1.0	2	95.98	158.14
21	2	2010_06	5.0	1	0.00	0.00
21	2	2010_06	5.0	2	0.00	0.00
21	2	2010_06	10.0	1	0.00	0.00
21	2	2010_06	10.0	2	1.45	0.02
21	2	2010_06	A	1	1202.67	3494.15
21	2	2010_06	A	2	1180.03	3600.77
21	2	2010_08	0.0	1	1300.50	4216.40
21	2	2010_08	0.0	2	735.48	2254.73
21	2	2010_08	0.1	1	154.11	283.23
21	2	2010_08	0.1	2	126.36	195.82
21	2	2010_08	1.0	1	65.04	80.28
21	2	2010_08	1.0	2	47.88	54.11
21	2	2010_08	5.0	1	2.01	0.00
21	2	2010_08	5.0	2	0.33	0.01
21	2	2010_08	10.0	1	0.00	0.00
21	2	2010_08	10.0	2	0.00	0.00
21	2	2010_08	A	1	1533.42	4858.33
21	2	2010_08	A	2	1587.88	4911.60
21	2	2010_10	0.0	1	915.88	2864.31
21	2	2010_10	0.0	2	1623.09	5348.21
21	2	2010_10	0.1	1	138.58	210.73
21	2	2010_10	0.1	2	124.27	211.76
21	2	2010_10	1.0	1	51.03	50.27
21	2	2010_10	1.0	2	76.05	98.52
21	2	2010_10	5.0	1	27.79	39.37
21	2	2010_10	5.0	2	14.66	6.88
21	2	2010_10	10.0	1	0.00	0.00

21	2	2010_10	10.0	2	0.00	0.00
21	2	2010_10	A	1	1431.27	4880.60
21	2	2010_10	A	2	826.16	2516.96
21	2	2011_02	0.0	1	1698.42	5977.52
21	2	2011_02	0.0	2	1944.65	6399.95
21	2	2011_02	0.1	1	188.07	419.82
21	2	2011_02	0.1	2	147.18	268.22
21	2	2011_02	1.0	1	83.78	80.91
21	2	2011_02	1.0	2	65.22	80.91
21	2	2011_02	5.0	1	3.24	0.15
21	2	2011_02	5.0	2	0.00	0.00
21	2	2011_02	10.0	1	0.00	0.00
21	2	2011_02	10.0	2	0.08	0.27
21	2	2011_02	A	1	2005.72	6737.51
21	2	2011_02	A	2	1937.43	6664.96
21	2	2011_15	0.0	1	1464.06	4724.60
21	2	2011_15	0.0	2	1368.25	4859.57
21	2	2011_15	0.1	1	141.92	204.09
21	2	2011_15	0.1	2	127.11	192.85
21	2	2011_15	1.0	1	39.55	64.47
21	2	2011_15	1.0	2	59.17	64.47
21	2	2011_15	5.0	1	0.00	0.00
21	2	2011_15	5.0	2	0.00	0.00
21	2	2011_15	10.0	1	0.33	0.10
21	2	2011_15	10.0	2	0.81	0.00
21	2	2011_15	A	1	1567.77	5393.67
21	2	2011_15	A	2	1035.54	3051.29
21	2	2011_63	0.0	1	1401.66	4586.74
21	2	2011_63	0.0	2	1267.76	3778.40
21	2	2011_63	0.1	1	195.64	345.37
21	2	2011_63	0.1	2	215.05	453.90
21	2	2011_63	1.0	1	90.20	152.62
21	2	2011_63	1.0	2	119.45	246.61
21	2	2011_63	5.0	1	21.61	16.26
21	2	2011_63	5.0	2	8.62	4.41
21	2	2011_63	10.0	1	0.00	0.00
21	2	2011_63	10.0	2	0.78	0.05
21	2	2011_63	A	1	1406.18	4942.71
21	2	2011_63	A	2	1379.79	4816.42
21	2	2011_67	0.0	1	1312.55	3857.25
21	2	2011_67	0.0	2	1323.96	4391.98

21	2	2011_67	0.1	1	162.41	314.47
21	2	2011_67	0.1	2	159.33	310.40
21	2	2011_67	1.0	1	106.16	174.13
21	2	2011_67	1.0	2	91.79	119.40
21	2	2011_67	5.0	1	0.00	0.00
21	2	2011_67	5.0	2	0.00	0.00
21	2	2011_67	10.0	1	0.00	0.00
21	2	2011_67	10.0	2	0.40	0.00
21	2	2011_67	A	1	1081.90	2815.19
21	2	2011_67	A	2	1819.24	6489.60
21	2	2011_88	0.0	1	2401.38	8700.30
21	2	2011_88	0.0	2	2326.85	7596.94
21	2	2011_88	0.1	1	100.17	131.31
21	2	2011_88	0.1	2	103.00	146.98
21	2	2011_88	1.0	1	54.54	66.91
21	2	2011_88	1.0	2	66.97	94.17
21	2	2011_88	5.0	1	0.00	0.00
21	2	2011_88	5.0	2	0.00	0.00
21	2	2011_88	10.0	1	1.03	0.12
21	2	2011_88	10.0	2	0.00	0.00
21	2	2011_88	A	1	1931.39	6496.74
21	2	2011_88	A	2	2274.06	7649.56
21	2	2012_051	0.0	1	1176.97	3723.04
21	2	2012_051	0.0	2	1154.08	3847.35
21	2	2012_051	0.1	1	155.29	298.34
21	2	2012_051	0.1	2	140.34	280.55
21	2	2012_051	1.0	1	61.06	68.08
21	2	2012_051	1.0	2	60.17	95.03
21	2	2012_051	5.0	1	2.72	0.25
21	2	2012_051	5.0	2	0.00	0.00
21	2	2012_051	10.0	1	0.00	0.00
21	2	2012_051	10.0	2	1.50	0.00
21	2	2012_051	A	1	1502.17	4848.46
21	2	2012_051	A	2	1519.91	4452.10
21	2	2012_086	0.0	1	1593.38	4734.36
21	2	2012_086	0.0	2	.	.
21	2	2012_086	0.1	1	120.70	163.77
21	2	2012_086	0.1	2	161.84	263.60
21	2	2012_086	1.0	1	68.59	93.48
21	2	2012_086	1.0	2	.	.
21	2	2012_086	5.0	1	7.03	2.14

21	2	2012_086	5.0	2	.	.
21	2	2012_086	10.0	1	2.50	0.00
21	2	2012_086	10.0	2	.	.
21	2	2012_086	A	1	1315.92	4198.01
21	2	2012_086	A	2	.	.
21	2	2012_117	0.0	1	1615.71	6407.04
21	2	2012_117	0.0	2	1187.72	4368.52
21	2	2012_117	0.1	1	112.98	175.54
21	2	2012_117	0.1	2	98.74	163.99
21	2	2012_117	1.0	1	56.97	84.95
21	2	2012_117	1.0	2	55.86	55.29
21	2	2012_117	5.0	1	22.88	16.33
21	2	2012_117	5.0	2	25.11	29.80
21	2	2012_117	10.0	1	0.00	0.00
21	2	2012_117	10.0	2	0.00	0.00
21	2	2012_117	A	1	1826.86	6209.96
21	2	2012_117	A	2	1415.10	4859.57
21	2	2012_125	0.0	1	1001.77	3159.94
21	2	2012_125	0.0	2	.	.
21	2	2012_125	0.1	1	147.79	270.55
21	2	2012_125	0.1	2	145.76	233.98
21	2	2012_125	1.0	1	81.01	108.43
21	2	2012_125	1.0	2	90.43	131.31
21	2	2012_125	5.0	1	1.24	0.00
21	2	2012_125	5.0	2	0.00	0.00
21	2	2012_125	10.0	1	0.08	0.01
21	2	2012_125	10.0	2	0.81	0.03
21	2	2012_125	A	1	787.11	2026.83
21	2	2012_125	A	2	1188.67	3987.12
21	2	2012_135	0.0	1	662.65	1811.07
21	2	2012_135	0.0	2	.	.
21	2	2012_135	0.1	1	121.92	232.89
21	2	2012_135	0.1	2	91.29	154.38
21	2	2012_135	1.0	1	55.26	61.51
21	2	2012_135	1.0	2	.	.
21	2	2012_135	5.0	1	32.91	20.11
21	2	2012_135	5.0	2	.	.
21	2	2012_135	10.0	1	0.57	0.32
21	2	2012_135	10.0	2	.	.
21	2	2012_135	A	1	753.07	2268.22
21	2	2012_135	A	2	.	.

21	2	2013_01	0.0	1	1549.81	5181.03
21	2	2013_01	0.0	2	1359.02	4044.41
21	2	2013_01	0.1	1	105.34	178.60
21	2	2013_01	0.1	2	171.41	293.16
21	2	2013_01	1.0	1	61.51	76.98
21	2	2013_01	1.0	2	58.79	65.61
21	2	2013_01	5.0	1	0.00	0.00
21	2	2013_01	5.0	2	0.08	0.04
21	2	2013_01	10.0	1	0.00	0.00
21	2	2013_01	10.0	2	0.79	0.00
21	2	2013_01	A	1	916.74	3059.13
21	2	2013_01	A	2	1192.63	3561.53
21	2	2013_03	0.0	1	1912.22	6768.10
21	2	2013_03	0.0	2	.	.
21	2	2013_03	0.1	1	135.22	256.17
21	2	2013_03	0.1	2	133.07	219.30
21	2	2013_03	1.0	1	119.48	193.84
21	2	2013_03	1.0	2	59.39	76.20
21	2	2013_03	5.0	1	33.64	28.56
21	2	2013_03	5.0	2	29.47	27.81
21	2	2013_03	10.0	1	0.00	0.00
21	2	2013_03	10.0	2	.	.
21	2	2013_03	A	1	1667.59	5239.88
21	2	2013_03	A	2	.	.
21	2	2013_04	0.0	1	2363.18	8450.82
21	2	2013_04	0.0	2	2033.05	7047.98
21	2	2013_04	0.1	1	176.26	366.44
21	2	2013_04	0.1	2	.	.
21	2	2013_04	1.0	1	110.53	186.75
21	2	2013_04	1.0	2	104.10	149.79
21	2	2013_04	5.0	1	0.00	0.00
21	2	2013_04	5.0	2	0.53	0.01
21	2	2013_04	10.0	1	1.51	0.04
21	2	2013_04	10.0	2	2.01	0.03
21	2	2013_04	A	1	1738.48	5920.11
21	2	2013_04	A	2	1512.17	5197.63
21	2	2013_11	0.0	1	1169.15	3893.66
21	2	2013_11	0.0	2	1289.61	4235.99
21	2	2013_11	0.1	1	153.78	216.16
21	2	2013_11	0.1	2	149.58	246.61
21	2	2013_11	1.0	1	97.49	133.14

21	2	2013_11	1.0	2	108.30	202.32
21	2	2013_11	5.0	1	0.00	0.00
21	2	2013_11	5.0	2	0.00	0.00
21	2	2013_11	10.0	1	0.00	0.00
21	2	2013_11	10.0	2	0.00	0.00
21	2	2013_11	A	1	974.42	3429.49
21	2	2013_11	A	2	1751.71	5658.49
21	2	2013_18	0.0	1	1582.31	5006.46
21	2	2013_18	0.0	2	645.79	1830.73
21	2	2013_18	0.1	1	144.31	278.18
21	2	2013_18	0.1	2	142.87	251.37
21	2	2013_18	1.0	1	105.46	163.99
21	2	2013_18	1.0	2	101.78	148.06
21	2	2013_18	5.0	1	16.15	5.23
21	2	2013_18	5.0	2	16.31	13.14
21	2	2013_18	10.0	1	0.00	0.00
21	2	2013_18	10.0	2	4.69	0.06
21	2	2013_18	A	1	1025.33	3320.35
21	2	2013_18	A	2	1305.83	4120.28
21	2	2013_20	0.0	1	902.03	2614.82
21	2	2013_20	0.0	2	683.52	1917.43
21	2	2013_20	0.1	1	186.96	306.35
21	2	2013_20	0.1	2	166.79	274.65
21	2	2013_20	1.0	1	105.67	151.75
21	2	2013_20	1.0	2	112.46	163.99
21	2	2013_20	5.0	1	1.24	0.04
21	2	2013_20	5.0	2	0.00	0.00
21	2	2013_20	10.0	1	0.00	0.00
21	2	2013_20	10.0	2	2.48	0.00
21	2	2013_20	A	1	1417.97	4432.01
21	2	2013_20	A	2	824.80	2777.70
